# PASTMUS: mapping functional elements at single amino acid resolution in human cells

**DOI:** 10.1186/s13059-019-1897-7

**Published:** 2019-12-16

**Authors:** Xinyi Zhang, Di Yue, Yinan Wang, Yuexin Zhou, Ying Liu, Yeting Qiu, Feng Tian, Ying Yu, Zhuo Zhou, Wensheng Wei

**Affiliations:** 1 Biomedical Pioneering Innovation Center, Beijing Advanced Innovation Center for Genomics, Peking-Tsinghua Center for Life Sciences, Peking University Genome Editing Research Center, State Key Laboratory of Protein and Plant Gene Research, School of Life Sciences, Peking University, Beijing, 100871 China; 2 Academy for Advanced Interdisciplinary Studies, Peking University, Beijing, 100871 China

## Abstract

Identification of functional elements for a protein of interest is important for achieving a mechanistic understanding. However, it remains cumbersome to assess each and every amino acid of a given protein in relevance to its functional significance. Here, we report a strategy, PArsing fragmented DNA Sequences from CRISPR Tiling MUtagenesis Screening (PASTMUS), which provides a streamlined workflow and a bioinformatics pipeline to identify critical amino acids of proteins in their native biological contexts. Using this approach, we map six proteins—three bacterial toxin receptors and three cancer drug targets, and acquire their corresponding functional maps at amino acid resolution.

## Background

RNA-guided CRISPR-associated protein 9 nucleases can introduce indels (insertions or deletions) and point mutations at target genomic loci by generating DNA double-strand breaks (DSBs) and consequently activating internal repair mechanisms, especially non-homologous end-joining (NHEJ) [1, 2]. Mutagenesis, and mutations leading to a frameshift in particular, can usually abolish protein expression, making the CRISPR-Cas9 system a powerful tool for genome engineering [3, 4] and even for high-throughput functional screening [5–8]. To better understand the role of regulatory elements or protein-coding sequences, CRISPR-mediated tiling mutagenesis has been employed with relevant biological assays [9, 10].

It is of great importance for the identification of functional elements for a protein of interest to achieve a mechanistic understanding. Traditional methods mainly rely on in vitro biochemical assays, such as co-immunoprecipitation (Co-IP) combined with truncation mutagenesis [11]; however, these techniques have a low resolution, and none of them is performed in native biological contexts. Previous studies include screening of cells expressing cDNAs containing various missense mutations [12, 13], screening through generating point mutations [14, 15], screening of tiling library followed by NGS (next-generation sequencing) on enriched sgRNAs [16–20], and a recent approach named “tag-mutate-enrich” [21]. Most of these methods require the exogenous expression of cDNAs [12, 13, 21]. They are also limited by the coverage of the actual amino acids of target [12–15, 21], the types of mutation [12–15], or the resolution of the functional map [16–20]. After all, most of these methods are not designed to study mutations that are genetically recessive [12, 13, 16–21]. There is no existing method that could assess potentially all amino acids of a given protein for their functional importance, especially in the native biological contexts.

Herein, we report the development of the PArsing fragmented DNA Sequences from CRISPR Tiling MUtagenesis Screening (PASTMUS) strategy, aiming at precisely mapping functional elements and assessing the importance of each amino acid (a.a.) spanning the full length of the protein of interest.

## Results

### Rationale, workflow, and bioinformatics pipeline of PASTMUS

**Fig. 1:**
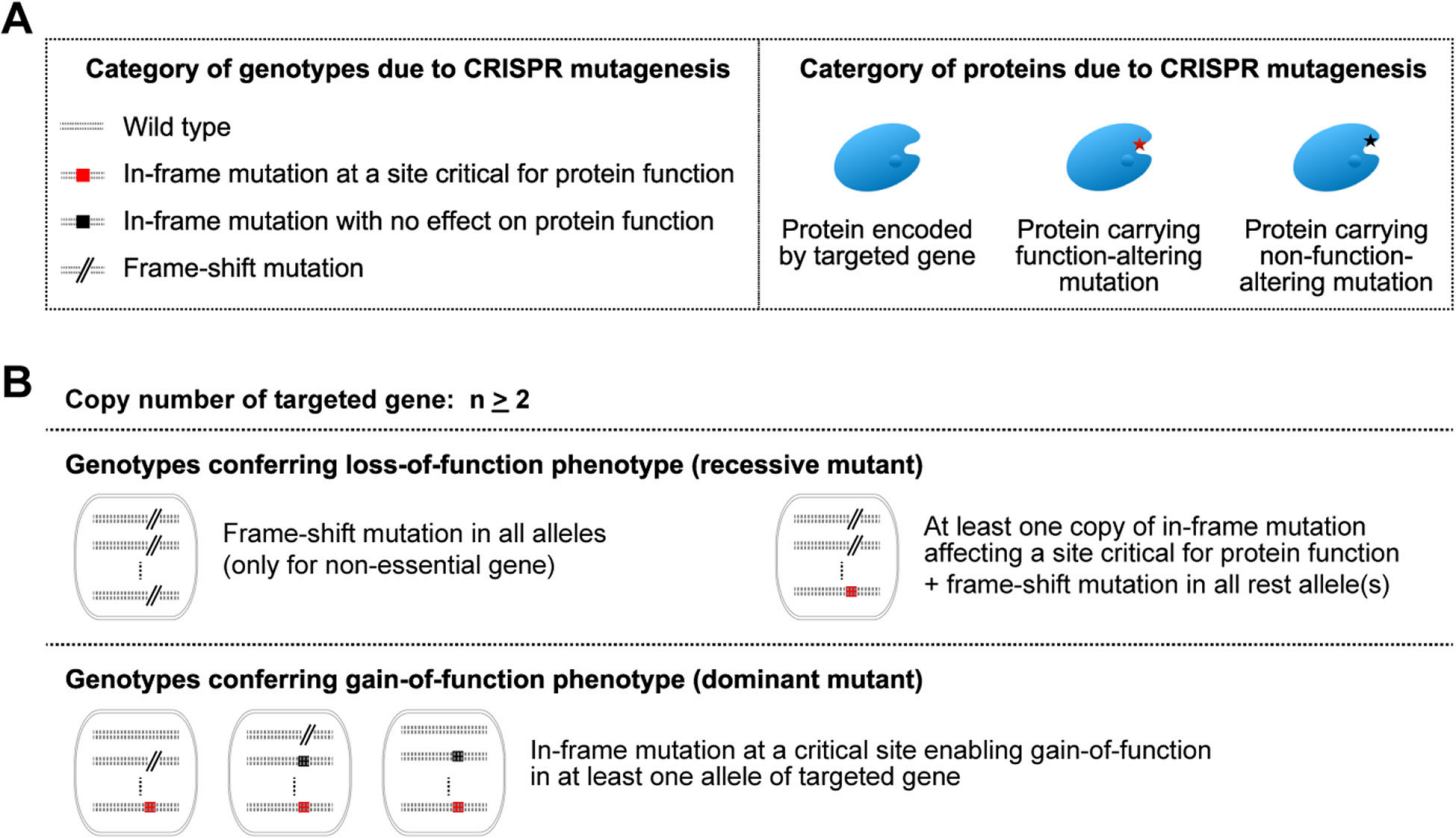
Rationale for acquiring residues critical for protein function based on phenotypic changes associated with in-frame mutations. **a** Category of genotypes or proteins due to CRISPR mutagenesis. **b** Genotypes conferring loss-of-function phenotype (for recessive mutant) and genotypes conferring gain-of-function phenotype (dominant mutant)

If we would generate a library of cells containing a variety of mutations spanning the targeted gene on the genome, we could readily enrich those cells harboring proteins carrying function-altering mutations in a positive selection screening (Fig. 1a). If mutations in targeted gene are genetically recessive, cells would have complete loss of function only if (i) frameshift mutations occur in all alleles (only for non-essential genes), or (ii) in-frame mutation affecting a site critical for protein function occurs in one or more allele(s), and frameshift mutation(s) in all the rest allele(s) (Fig. 1b, Additional file 1: Figure S1). For the genetically dominant mutant, in-frame mutation at a critical site enabling gain-of-function phenotype in at least one allele of targeted gene is sufficient to confer phenotypic change (Fig. 1b, Additional file 1: Figure S1). We therefore hypothesized that if we were to apply CRISPR tiling mutagenesis and retrieve only in-frame mutations (in-frame deletions or missense mutations) that give rise to a phenotypic change of choice, we could identify critical amino acids relevant to the protein functions.

**Fig. 2:**
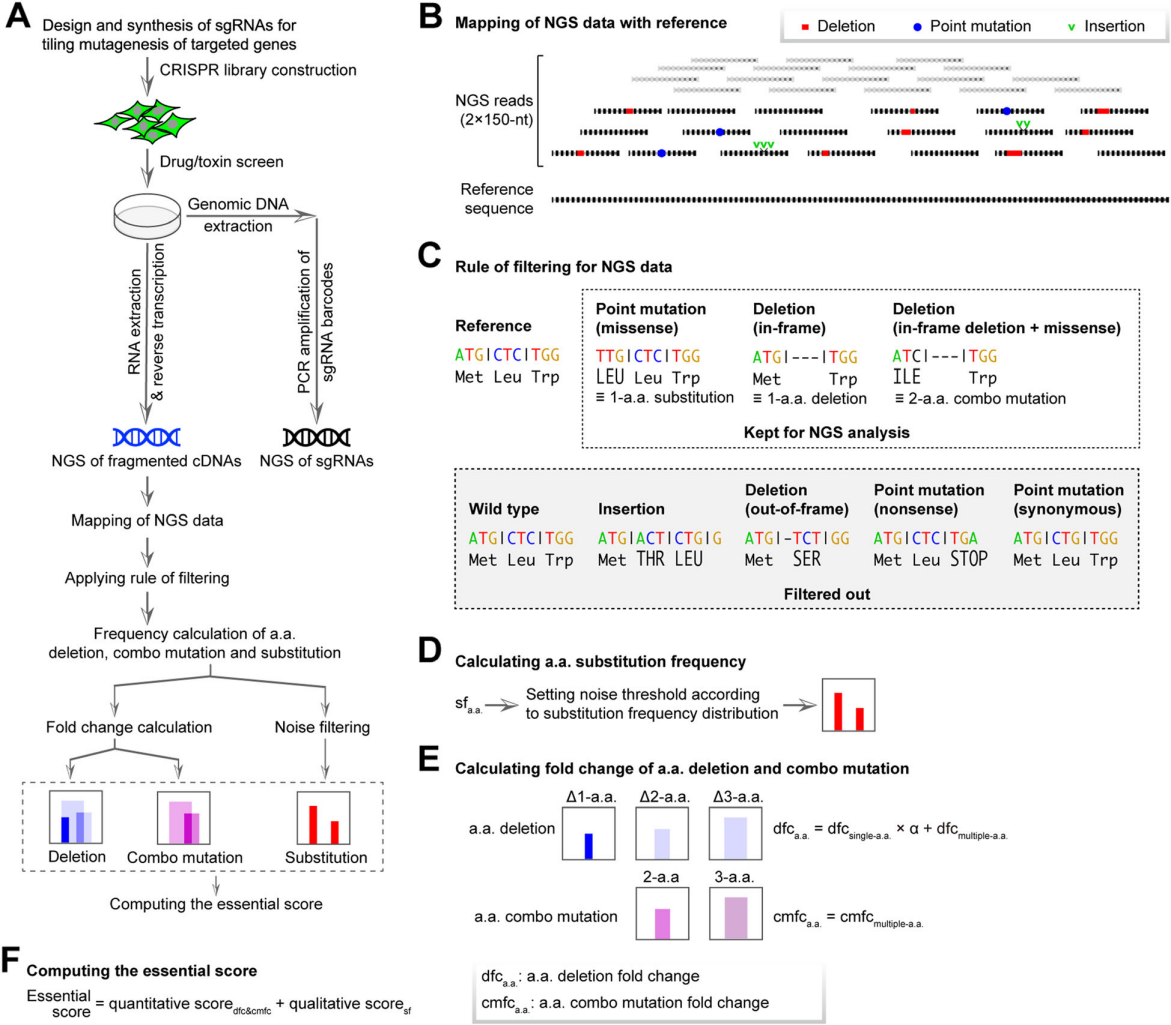
PASTMUS workflow and bioinformatics pipeline to identify critical residues of proteins. **a** PASTMUS workflow. sgRNA tiling library screening was performed, followed by amplification of sgRNA barcodes and the targeted gene cDNA for NGS. After mapping to targeted gene CDS and extracting mutation, sequencing reads with out-of-frame mutations were filtered out. The frequency of reads conferring amino acid deletion, combo mutation, and substitution were calculated for libraries before and after screening. Quantitative assessment (fold change) of a.a. deletions and combo mutations and qualitative assessment of a.a. substitutions were performed, respectively. **b–f** The essential scores of individual amino acids indicating their functional significance were calculated accordingly. The PASTMUS bioinformatics pipeline is as follows: mapping of NGS data with reference sequence (**b**), applying the filtering rules for NGS data (**c**), calculating a.a. substitution frequency and setting noise threshold for filtering (**d**), calculating fold change of a.a. deletion and a.a. combo mutation between libraries before and after screening (**e**), and computing essential scores for individual a.a. according to quantitative assessment of a.a. deletion and a.a. combo mutation, and qualitative assessment of substitution frequency (**f**)

We first performed tiling mutagenesis of targeted genes using the CRISPR-spCas9 system [8, 22, 23]. To maximize the coverage density in designing sgRNAs, we included two types of protospacer-adjacent motifs (PAMs), NGG and NAG [24]. After library screening using bacterial toxins or cancer drugs, genomic DNA was extracted for the conventional PCR amplification of sgRNA barcodes, followed by NGS analysis. In addition, cDNAs obtained from reverse-transcribed RNAs of targeted genes were PCR amplified and subsequently fragmented to approximately 250 bp in length before subjected to NGS. NGS data have to be mapped with reference and applied with a series of rules to obtain frequencies of those “meaningful” mutant reads at functionally relevant sites (Fig. 2a).

To determine whether we could generate sufficient mutation variants for PASTMUS and how the sgRNA coverage (cell count per sgRNA) corresponds to mutation complexity, we performed CRISPR mutagenesis on eight sites from *CSPG4* and *HBEGF* genes (Additional file 1: Figure S2, Additional file 2: Table S1). With variable folds of sgRNA coverages, total mutations and in-frame mutation types were calculated based on NGS (at average base coverage of sequencing ~ 50,000×). Wild type loci were also sequenced following the same experimental protocol to determine the basal level of mutations due to PCR and sequencing errors. It turned out that the types of in-frame mutants, as well as the total mutants after CRISPR mutagenesis in all eight sites, were significantly higher than the basal levels. It is also evident that the higher the sgRNA coverage, the larger the mutant varieties. It is therefore beneficial to generate a sgRNA library with as high as possible the coverage to maximize mutant complexity for screening (Additional file 1: Figure S2).

To test PASTMUS strategy in mapping functional elements of proteins, we selected three genes (*ANTXR1*, *CSPG4*, and *HBEGF*) encoding bacterial toxin receptors and three genes (*HPRT1*, *PLK1*, and *PSMB5*) encoding cancer drug targets (Additional file 3: Table S2). We chose HeLa cells to construct the CRISPR library for screening because we have determined the appropriate killing conditions in this cell line for toxins [8, 11, 25] and drugs (e.g., 6-TG targeting HPRT1 [26], BI2536 targeting PLK1 [27], and Bortezomib targeting PSMB5 [28]) (Additional file 1: Figure S3).

For the targeted genes, sgRNAs were designed in silico and synthesized on a chip as pools to construct a tiling CRISPR library covering the full lengths of the three receptor-coding genes and a library covering three drug targets (Additional file 1: Figure S3, Additional file 4: Table S3, Additional file 5: Table S4). We performed functional screens in two replicates for each of the six treatments in addition to controls without treatment. After three rounds of treatment with a toxin (PA/LFnDTA toxin, diphtheria toxin, or *Clostridium difficile* toxin B) or a drug (6-TG, BI2536, or Bortezomib), resistant cells were harvested, and genomic DNA was extracted for conventional sgRNA deciphering through NGS analysis [8, 29]. The harvested resistant cells were also subjected to total RNA isolation and reverse transcription to obtain cDNAs, which were subsequently used as templates for PCR amplification using specific primers (Additional file 5: Table S5). For genes with big sizes, such as *CSPG4*, multiple pairs of primers were used to amplify overlapping fragments to encompass their full lengths. For genes with alternative splicing, specific primer pairs were designed to ensure that all alternative transcripts were included (Additional file 1: Figure S3).

To meet the size limitation for NGS, PCR amplification of cDNA was fragmented to average 250 bp (Fig. 2a, Additional file 1: Figure S3). Since the mixtures of DNA fragments were predominantly wild type sequences, it is critical to reach enough sequencing depths to identify those small percentages of mutants. For this, we have developed a bioinformatics pipeline and applied a series of filtering procedure to process NGS data (Fig. 2). Among all types of mutations, deletion, insertion, and point mutation (Fig. 2b), we only kept those falling into one of the following two categories: missense mutation leading to amino acid substitution, and in-frame deletion leading to either a.a. deletion or a.a. combo mutation (due to the combined effect of in-frame deletion and missense mutation) (Fig. 2c). All wild type genes of targets were also sequenced following the same experimental protocol to determine the basal level of mutations.

From six gene-targeting PASTMUS libraries before screening, the levels of in-frame deletions leading to either a.a. deletions or a.a. combo mutations were significantly higher than the mock controls (Additional file 1: Figure S4); however, the levels of missense mutations leading to a.a. substitutions were indistinguishable between libraries and the mock controls (Additional file 1: Figure S4). Several recent studies [30, 31] reported that substitution frequency generated by DNA repair after CRISPR-spCas9 editing is relatively low, while the errors generated through the course of reverse transcription, PCR amplification, and NGS were predominantly point mutations rather than indels. Although we were unable to normalize missense mutation, the enrichment of such type through screening provided an affirmative answer for the functional importance of the affected amino acid (Fig. 2d).

For a.a. deletion and combo mutation types (Fig. 2c), more than 95% of amino acids encoded by the six targeted genes were covered. In particular, 98% of amino acids were covered for genes with relatively smaller sizes when counting mutations affecting up to three a.a., e.g., *HBEGF* and *HPRT1* (Additional file 1: Figure S5). To reduce potential false-positive rate, we counted only those mutations affecting ≤ 3 a.a. for further analysis. The enrichment of a.a. deletions and combo mutations could be benchmarked by fold changes with their frequencies in the original libraries before the screening. Since the affirmative role of any given amino acid could be determined by single-a.a. deletion result, we assigned a full weight to those sites and a discounted weight (based on mutation lengths) to those with multiple-a.a. deletion or combo mutation (Fig. 2e and the “Methods” section). Combining both the quantitative data for a.a. deletion or combo mutation and the qualitative data for a.a. substitution, we could compute the essential scores to obtain the importance of all amino acids in relevance to protein function (Fig. 2f).

**Fig. 3:**
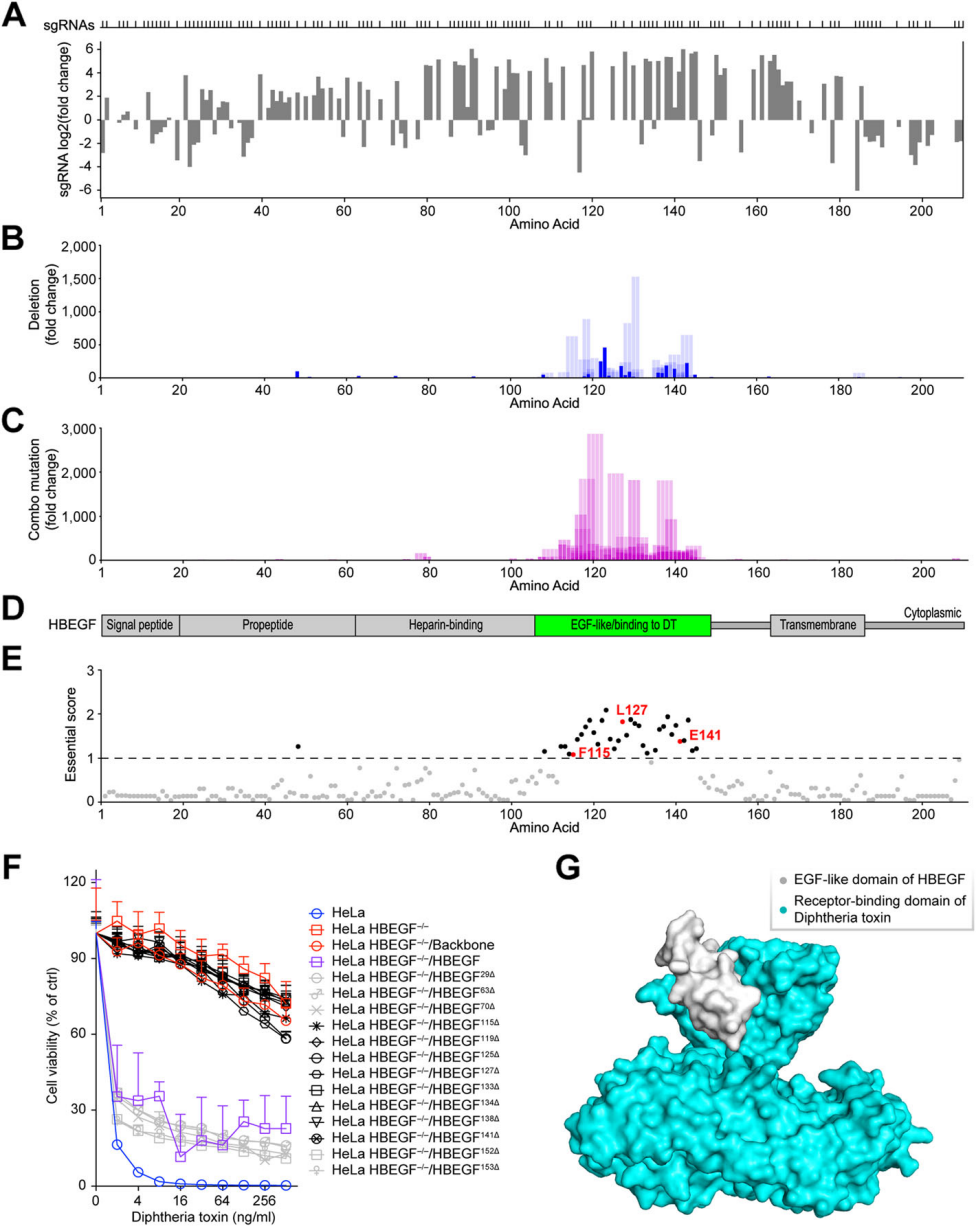
Identification of HBEGF amino acids critical for diphtheria toxin (DT)-mediated cytotoxicity through PASTMUS. **a** Identification of *HBEGF*-targeting sgRNAs conferring cell resistance to DT. Distribution of sgRNAs mapped to the corresponding amino acid in HBEGF is indicated on top. **b** a.a. deletion fold change corresponding to each amino acid. The solid blue bars indicate single-a.a. deletions; bars with transparency indicate multiple-a.a. deletions. The width of the blue bar with transparency corresponds with a.a. deletion length. **c** a.a. combo mutation fold change corresponding to each a.a. Width of the bar indicates affected a.a. length. **d** Schematic diagram of HBEGF with the EGF-like domain shown in green, a known binding region for DT. **e** Essential score of each a.a. of HBEGF. Cutoff of essential score is plotted as a dashed line, with critical amino acids above the cutoff shown in black and known critical amino acids labeled in red. **f** Effects of single-a.a. deletions on the susceptibility of cells to DT. Cells were treated with different concentrations of DT, and the MTT cytotoxicity assay was performed 48 h after toxin treatment. Data are presented as the mean ± SD, *n* = 5. **g** Surface diagram of crystal structure of the complex of DT with EGF-like domain of HBEGF. The EGF-like domain of HBEGF is shown in gray, and the receptor-binding domain of DT is shown in cyan (PDB code: 1XDT)

We evaluated the quality of the screens based on the sgRNA fold changes between two replicates and obtained the correlation coefficients ranging from 0.71 to 0.98 (Additional file 1: Figure S6). Because all three toxin receptors are non-essential for cell viability, the sgRNAs observed after screening were uniformly distributed across their coding sequences (Fig. 3a, Additional file 1: Figures S8, S9), indicating that most of them could generate frameshift indels, resulting in the disruption of targeted protein expression. However, NGS of sgRNA-coding regions revealed little sequence-to-function information.

**Fig. 4:**
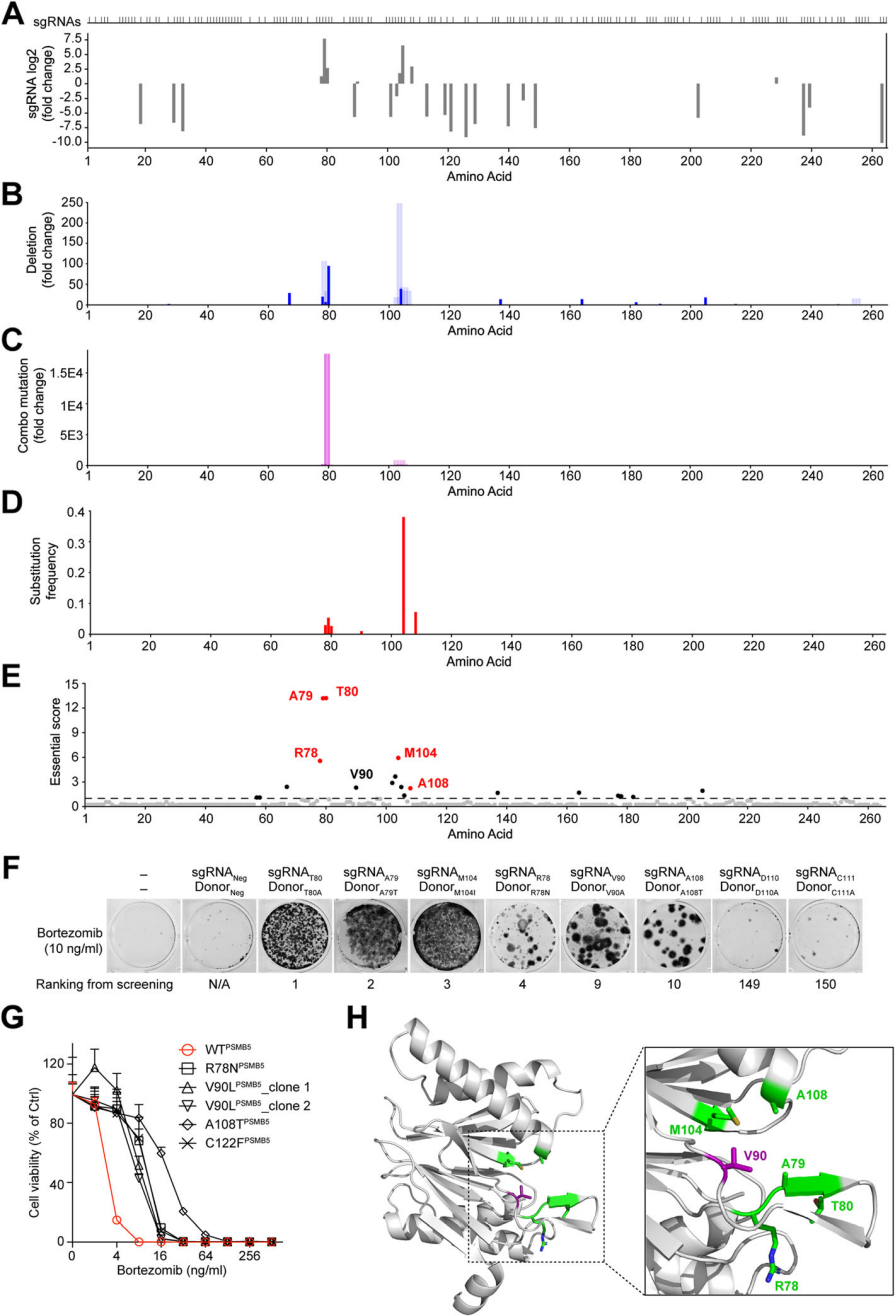
Identification of PSMB5 amino acids critical for Bortezomib-mediated killing through PASTMUS. **a** Identification of *PSMB5*-targeting sgRNAs conferring cell resistance to Bortezomib. Distribution of sgRNAs mapped to the corresponding amino acid in PSMB5 is indicated on top. **b** a.a. deletion fold change corresponding to each a.a. **c** a.a. combo mutation fold change corresponding to each a.a. **d** a.a. substitution frequency corresponding to each a.a. **e** Essential score of each a.a. of PSMB5. Cutoff of essential score is plotted as a dashed line, with critical amino acids above the cutoff shown in black and known critical amino acids labeled in red. **f** MTT viability assay for indicated substitutions of PSMB5 on the susceptibility of cells to Bortezomib. **g** Effects of indicated substitutions in PSMB5 on the susceptibility of cells to Bortezomib. Data are presented as the mean ± SD, *n* = 6. **h** Crystal structure of PSMB5. Five positive amino acids and the novel critical site, V90, are labeled in green and purple, respectively (PDB code: 5LF3)

By applying PASTMUS with the computational pipeline, we obtained function-related amino acid maps. We purposely assigned a solid blue color to single-a.a. deletions because there is no ambiguity regarding the significance of such mutations, while we assigned blue with 15% transparency to multi-a.a. deletions (Figs. 3b and 4b, Additional file 1: Figures S8, S9, S10, S11), purple for a.a. combo mutations with higher transparent levels set for longer affected length (Figs. 3c and 4c, Additional file 1: Figures S8, S9, S10, S11), and red for the a.a. substitutions (Fig. 4d, Additional file 1: Figures S9, S10, S11).

### Mapping toxin receptors

For the functional screening of *HBEGF*, which encodes a receptor for diphtheria toxin (DT), most of the resistant cells carried a.a. deletions and combo mutations in the EGF-like domain (Fig. 3b–d), a reported DT-binding site [32]. Essential scores (Fig. 3e, Additional file 6: Table S6) indicated that the amino acids with the highest scores were enriched in the EGF-like domain, further confirming the essentiality of this domain for mediating toxin binding [32]. Three amino acids, F115, L127, and E141 [32], that are known to be essential for the HBEGF-DT interactions were ranked at the top (35th, 7th, and 22nd) among all amino acids. Importantly, PASTMUS uncovered a number of novel sites in addition to these three that appeared important for receptor function (Fig. 3e). To validate these results, we expressed wild type and mutant *HBEGF* from cDNAs in HeLa *HBEGF*^−/−^ cells [8] via lentiviral infection (Additional file 1: Figure S7, Additional file 5: Table S7). We verified five top-ranking sites (G119, K125, I133, C134, and Y138), three known positive sites (F115, L127, and E141), and five other sites below the threshold (L29, D63, D70, N152, and R153). HeLa *HBEGF*^−/−^ cells appeared to be completely resistant to DT, and wild type *HBEGF* expression recovered cell sensitivity to the toxin. The expression of all mutant forms of HBEGF with a single-a.a. deletion in one of these five top-ranking sites (G119, K125, I133, C134, and Y138) or known positive sites (F115, L127, and E141) failed to rescue the sensitivity of cells to DT, while mutant HBEGF with single-a.a. deletion in one of five other sites (L29, D63, D70, N152, and R153) could make the rescue like wild type (Fig. 3f). Crystal structure of the complex of DT toxin with EGF-like domain of HBEGF illustrated the interaction between toxin and its receptor-binding site (Fig. 3g, Additional file 7: Video S1). Notably, the fact that very few amino acids outside of the DT-binding domain of HBEGF were screened out indicated a low false-positive rate of PASTMUS.

For the anthrax toxin receptor, ANTXR1, all resistant cells carried a variety of a.a. deletions and combo mutations across the entire coding region, including known sites corresponding to PA binding (Additional file 1: Figure S8). In addition to the known PA-binding sites [33] and transmembrane domain, a number of novel amino acids showing variable levels of importance were identified (Additional file 1: Figure S8). The most important amino acids for the function of ANTXR1 in mediating anthrax toxicity were determined by computing essential scores, including one known site, H57 [33] (Additional file 1: Figure S8).

For CSPG4, the receptor of *Clostridium difficile* toxin B (TcdB) [11], critical amino acids were mainly located in the first and last two CSPG repeats (Additional file 1: Figure S9). The first CSPG repeat is a known TcdB-binding site [11], while the last two repeats represented novel findings. Importantly, unlike the above two cases involving HBEGF and ANTXR1, in which the most informative data came from a.a. deletions and combo mutations, a.a. substitutions affecting Q780 in CSPG4 were highly enriched (in red, Additional file 1: Figure S9), suggesting a critical role of this residue in mediating TcdB toxicity.

### Mapping cancer drug targets

Regarding the three genes encoding cancer drug targets, *HPRT1* is a non-essential gene, while both *PLK1* and *PSMB5* are essential for cell viability [34]. For HPRT1, 6-TG screening of the library showed that most of the sgRNAs were enriched and evenly distributed (Additional file 1: Figure S10), similar to those in bacterial toxin screens (Fig. 3a, Additional file 1: Figures S8, S9). The significance of each amino acid throughout the protein was indiscernible from sgRNA sequencing analysis (Additional file 1: Figure S10). The PASTMUS approach revealed a great number of sites that appeared important for HPRT1 in mediating cell sensitivity to 6-TG (Additional file 1: Figure S10). These findings were consistent with the known structure of tetrameric HPRT1 [26] (Additional file 1: Figure S10).

For the essential targets, PLK1 and PSMB5, sgRNA sequencing provided the approximate locations of certain critical amino acids where sgRNAs generated in-frame mutations (Fig. 4a, Additional file 1: Figure S11). Because sgRNA enrichment provided indirect evidence with low resolution, we reasoned that PASTMUS strategy would provide a more precise and comprehensive map with enhanced details. Indeed, more amino acids that appeared critical for protein function were identified with high accuracy in both PSMB5 and PLK1 (Fig. 4b–d, Additional file 1: Figure S11). Notably, top essential amino acids were identified mainly from a.a. substitutions, complemented by a variable number of a.a. deletions and combo mutations (Fig. 4d, e, Additional file 1: Figure S11). We again identified both known critical sites in PSMB5 for its interaction with Bortezomib (R78, A79, T80, M104, and A108) [35–37] and novel essential residues such as V90 (Fig. 4d, e). Similarly, we identified the residues C67 and R136, which are known to be critical for the BI2536-PLK1 interaction [37, 38], as well as a novel essential residue F183 (Additional file 1: Figure S11).

Because a.a. substitution was the predominant mutant type conferring drug resistance for both PSMB5 and PLK1, we decided to employ the ssODN-mediated method [39] to generate specific substitutions, instead of a.a. deletions, for validation. We selected eight sites (R78, A79, T80, V90, M104, A108, D110, and C111) in PSMB5, among which D110 and C111 were bottom-ranked and served as controls. The mutant types from the screening results or previous reports were preferentially chosen for substitution in the validation experiments. For the remainder, alanine was used for substitution (Additional file 5: Table S8). The transfected cells with donors containing one of six substitutions (R78N, A79T, T80A, V90A, M104I, and A108T) produced a variable number of Bortezomib-resistant colonies (Fig. 4f). In comparison, D110A and C111A failed to produce Bortezomib resistance, demonstrating that our method of validation was reliable (Fig. 4f). Interestingly, the C111 site has previously been reported to be important for PSMB5 in SW1573 and CEM cells [36, 40], in contrast to our screening and validation results (Fig. 4f). This discrepancy suggests that either the role of this amino acid varies with biological context, or the mutation leading to the correct type of a.a. substitution was missing in the original library. To verify the Bortezomib-resistant cells, we sequenced the genomic region of the target loci and confirmed that all six sites contained the expected substitutions (Additional file 1: Figure S12, Additional file 5: Table S9). To further verify our results, we isolated single clones (Additional file 1: Figure S13) and performed the cell viability assay. We demonstrated that the following substitutions conferred Bortezomib resistance: R78N, V90L, and A108T (Fig. 4g). Among these substitutions, T80 and A108 have been previously reported to be involved in the direct binding of PSMB5 to Bortezomib [35–37], and substitutions of R78, A79, and M104 have been reported to disrupt the structures of the drug-binding sites and consequently confer Bortezomib resistance [14, 37, 41]. For the novel site, V90, we confirmed that V90L conferred drug resistance with two independent clones (Fig. 4g). Crystal structure of PSMB5 showed that V90 together with five known critical amino acids, R78, A79, T80, V90, M104, and A108, were all located in the pocket of PSMB5 that interacts with Bortezomib (Fig. 4h).

For PLK1, it has been reported that R136 and C67 are critical amino acids for BI2536 and F183 is structurally important for PLK1 binding to BI2536 [2, 37, 38]. A substitution in each of these three sites was confirmed to confer BI2536 resistance (Additional file 1: Figure S11).

### PASTMUS reveals substitution patterns for critical residues

**Fig. 5:**
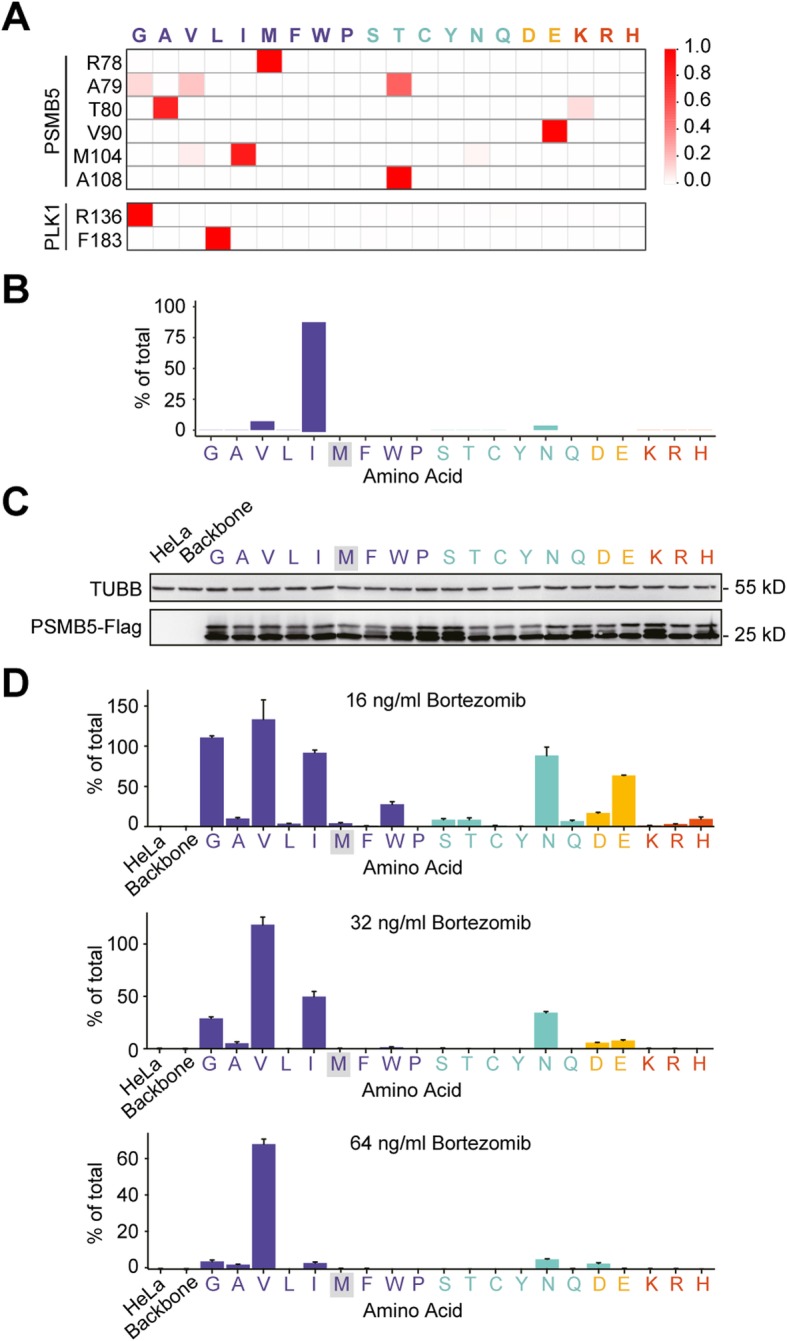
Substitution pattern analysis of top hits from PSMB5 and PLK1. **a** Heat maps showing the substitution pattern of top amino acids of PSMB5 and PLK1. The 20 amino acids are classified into 4 groups with different colors according to their side-chain properties: non-polar (purple), polar (aqua), acidic (yellow), and basic (red). **b** PSMB5 M104 substitution pattern enriched in PASTMUS. **c** The expression of all PSMB5 M104 substitution variants. **d** Effects of M104 substitution variants on Bortezomib-mediated cell cytotoxicity with indicated dosages. Data are presented as the mean ± SD, *n* = 3

Since each amino acid has 19 kinds of non-synonymous substitutions, we hypothesized that different substitutions might have distinct effects. We retrieved missense mutation data of top hits from PSMB5 and PLK1 and performed substitution pattern analysis. Indeed, there was a clear pattern preference of substitution for these amino acids to confer cell resistance to drugs (Fig. 5a). In the case of PSMB5 at the site M104, PASTMUS identified three substitution variants (M104V, M104I, and M104N) that conferred Bortezomib resistance (Fig. 5a, b). To determine whether these were the only 3 resistance-conferring substitutions and how powerful the PASTMUS strategy is in generating substitution variety, we expressed wild type and 19 kinds of PSMB5 M104 mutants in HeLa cells via lentiviral infection (Additional file 5: Table S10). Besides these three, several other substitution variants of M104 also conferred drug resistance. With a compatible exogenous expression of all substitution variants (Fig. 5c), M104V appeared much more resistant than many other variants, especially to high dosage of Bortezomib (Fig. 5d). Interestingly, PASTMUS identified that V90 had a preference in glutamate (E) that conferred Bortezomib resistance (Fig. 5a). We expressed wild type and PSMB5 V90E mutants in HeLa cells (Additional file 5: Table S10). With a comparable expression level of wild type (Additional file 1: Figure S14), V90E conferred significant resistance to Bortezomib (Additional file 1: Figure S14). Owing to the high possibility that multiple substitution variants at critical sites could confer drug resistance, we did not suffer from a high level of false discovery rate for PASTMUS, even though we could not generate all substitution types for a given site in the original tiling mutagenesis library.

### Sequencing depth in PASTMUS

Because in-frame deletions and missense mutations generated by tiling CRISPR mutagenesis comprised only small percentages of all sequenced fragments, we would like to determine the proper sequencing depth to detect these rare events. To this end, we performed subsampling to the reads of *HBEGF* and *PSMB5* genes at different sequencing coverage of libraries before and after screening (Additional file 1: Figures S15, S16). For original libraries before the screening, sequencing depth of 1.5E7× and 1E7× appeared sufficient for *HBEGF* and *PSMB5*, respectively (Additional file 1: Figure S15, S16). The difference between these two genes is likely because *PSMB5* is an essential gene, and its out-of-frame mutants cause cell death. After the positive screening, however, the required sequencing depth became 1E6×, much lower than the original library before screening (Additional file 1: Figure S15, S16). Hence, we would recommend a sequencing depth of 1.5E7× and 1E7× for original libraries before screening targeting non-essential and essential gene, respectively, and 1E6× for libraries post-screening.

### Structural superposition of protein functional maps

**Fig. 6:**
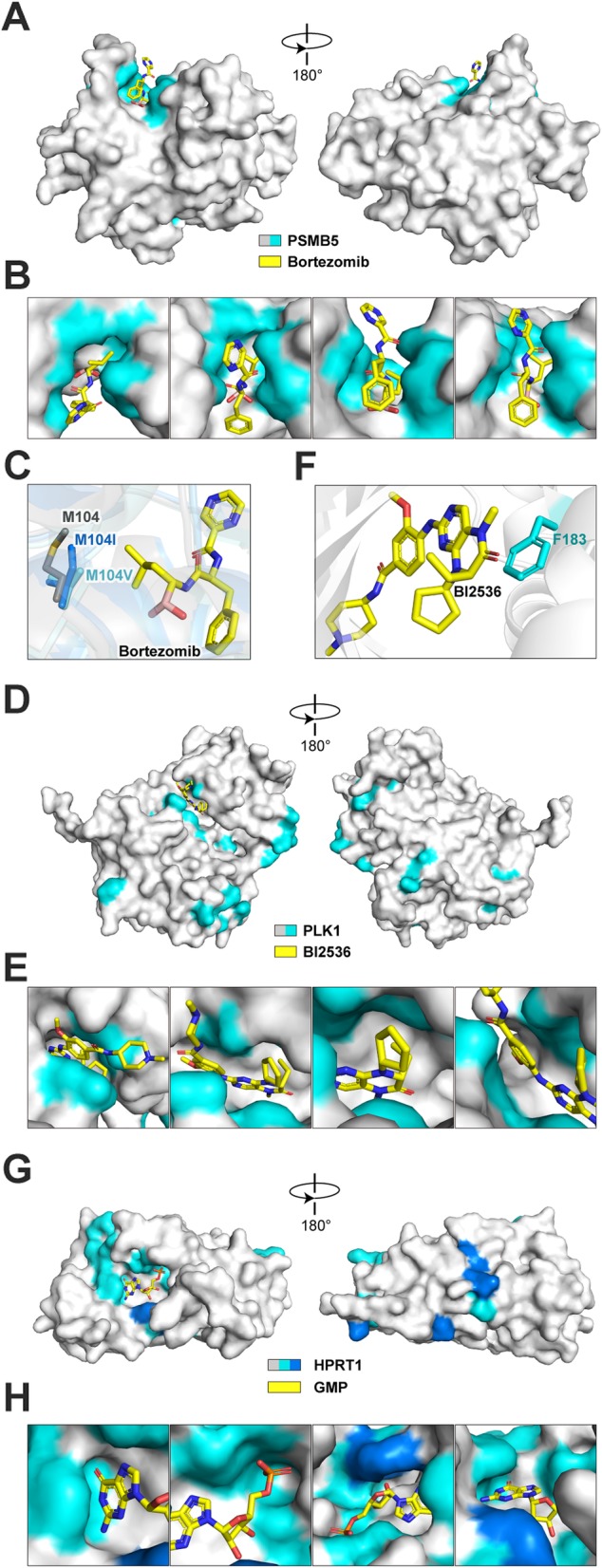
Functional maps of PSMB5, PLK1, and HPRT1 at structure level. **a** Surface diagram of PSMB5 with Bortezomib. PSMB5 is shown in gray, and critical sites enriched in PASTMUS are shown in cyan. Bortezomib is shown in yellow (PDB code: 5LF3). **b** Zoomed-in view shown the interface of PSMB5 and Bortezomib. **c** Structural comparison of PSMB5 M104, M104I, and M104V interacts with Bortezomib (PDB code: 2F16, 4QVN, and 4QVQ). **d** Surface diagram of PLK1 with BI2536. PLK1 is shown in gray, and critical sites are shown in cyan. BI2536 is shown in yellow (PDB code: 2RKU). **e** Zoomed-in view shown the interface of PLK1 and BI2536. **f** Interaction of PLK1 F183 and BI2536. **g** Surface diagram of HPRT1 with bound GMP. HPRT1 is shown in gray, and critical sites are shown in blue (critical sites known for dimer or tetramer interaction are colored dark blue) (PDB code: 1HMP). **h** Zoomed-in view shown the interface of HPRT1 and GMP

To compare the functional maps with their corresponding protein structures, we highlighted those critical residues identified from PASTMUS on the surface diagram of three cancer drug targets (PSMB5, PLK1, and HPRT1) (Fig. 6a, d, g). For PSMB5, functional mapping results at the linear sequence format revealed little information regarding the spatial correlations of those critical sites and the drug (Additional file 1: Figure S4); however, this became self-explanatory as most of those identified amino acids were located in the pocket embracing Bortezomib (Fig. 6a, b, Additional file 8: Video S2) in the 3D structure. Comparing the wild type PSMB5 and its M104 mutants, the slight changes of amino acid side chains for M104I and M104V (Fig. 6c) were likely responsible for weakened interaction of PSMB5 with Bortezomib, thereby resulting in drug resistance. M104V appeared to have much shorter side chain than M104 and M104I, and thus conferred Bortezomib resistance at a much higher dosage (Fig. 5d).

Similarly, for PLK1, most of the identified residues (L59, C67, R136, and N181-L184) are located within the pocket that binds to BI2536 (Fig. 6d, e, Additional file 9: Video S3). F183, a structurally important site [42], showed a direct interaction with BI2536 through π-π stacking between aromatic rings (Fig. 6f). F183L identified from PASTMUS might disrupt the π-π stacking, leading to drug resistance (Fig. 5a, Additional file 1: Figure S11). Notably, several critical amino acids identified are located outside of the binding pocket (Fig. 6d), suggesting that these types of mutations could remotely alter binding pocket. This is particularly important because such amino acids are hardly predictable from the crystal structure.

For HPRT1, a transferase catalyzing the conversion of guanine to guanine monophosphate and hypoxanthine to inosine monophosphate, its crystal structure bound with guanine monophosphate (GMP) showed that the identified amino acids were mostly housed within the pocket embracing GMP (Fig. 6g, h, Additional file 10: Video S4). This was consistent with the finding that 6-thioguanine (6-TG) acts as a purine analog to inhibit HPRT1 [26]. Moreover, several amino acids shown in dark blue were known sites essential for HPRT1 dimer or tetramer interaction. Mutations of these types of amino acids might disrupt HPRT1 tetramer formation, resulting in the loss of protein function and consequently the 6-TG resistance.

## Discussion

Although previous studies using tiling mutagenesis have been reported to identify critical residues of proteins of interest [16–44], PASTMUS strategy is different from all of them. Our method enabled the identification of functionally important sites of the protein of interest at its native biological context and could work for both dominant and recessive mutations, regardless of the target gene size. The use of truncation mutagenesis to identify potential functional domains is often laborious. It is also technically difficult, if not impossible, to assess the significance of every amino acid spanning the full length of the protein of interest. Gill and colleagues recently described a method for mapping functionally relevant mutations in a protein of interest in bacteria or yeast; however, this method relies heavily on the homologous recombination rate, preventing its effective application in higher eukaryotes [45]. Moreover, PASTMUS allows multiple genes to be scanned simultaneously to identify functional elements in their corresponding proteins.

PASTMUS could delineate a functional map of a protein. Importantly, PASTMUS reveals critical amino acids that are located in both “reasonable” and “unreasonable-appearing” sites based on protein’s structural data. Those residues out of the catalytic domain or drug-binding pocket may provide valuable information for in-depth mechanisms of function of the protein. In addition, when co-crystallization data are missing, PASTMUS might be helpful to determine the precise binding sites of small chemical compounds, or even help to calibrate protein structures.

Although PASTMUS is capable of generating abundant mutations on almost all amino acids across the target protein, the function-altering mutation does not necessarily indicate that the affected site is directly relevant to protein function. For non-essential genes, at least two types of mutations could be identified from PASTMUS. The first is the mutation on a site that is critical for protein function. The second type is the mutation on a site that is critical to maintain the overall protein conformation or structure. For instance, we identified many hits that were located within the transmembrane domain of ANTXR1 (Additional file 1: Figure S8), a region that is important for the presence of receptor on the cell surface, not necessarily directly involved in toxin endocytosis.

For gain-of-function mutations, the “hitchhiking effect” could be a possible source of false positive (Additional file 1: Figure S1). Among the results of six gene-targeting PASTMUS screening, we did not find out those kinds of false-positive sites. For adjacent amino acids that appear as hits in our PSMB5 screening, we could verify the importance of R78, A79, and T80. Thus, this “hitchhiking effect” would not be a severe problem in PASTMUS strategy. This is likely because that the frequency of any “meaningful” in-frame mutation is extremely low, which makes the case of two or more different in-frame mutation variants in the same cell a very rare incidence.

## Conclusions

We report a high-throughput strategy, PASTMUS, that provides a streamlined workflow and a bioinformatics pipeline for identifying critical elements of proteins in their native biological contexts. We mapped six proteins and acquired corresponding comprehensive functional maps at a single amino acid resolution; these maps contained both known domains or sites and novel amino acids that are critical for drug or toxin sensitivity. This method revealed comprehensive and precise single amino acid substitution patterns for critical residues. Because both a.a. deletions and combo mutations could be determined and quantified in the original libraries before the screening, PASTMUS could be readily applied in negative screening. Moreover, PASTMUS strategy is also suited for acquiring functional maps of regulatory elements, such as non-coding RNA, promoters, and enhancers.

## Methods

### Cells and reagents

Stably Cas9-expressing HeLa cells [8] and HEK293T cells were cultured in Dulbecco’s modified Eagle’s medium (DMEM, Corning) containing 10% fetal bovine serum (FBS, CellMax) under 5% CO_2_ at 37 °C. All cells were checked for the absence of mycoplasma contamination. STR analysis was used for cell line authentication.

### Plasmid construction

The sgRNA vector (pLenti-sgRNA-GFP) was cloned by replacing the U6 promoter in pLL3.7 (Addgene) with the human U6 promoter, ccdB cassette, and sgRNA scaffold. The Cas9 expression vector (pLenti-OC-IRES-BSD) has been previously reported [8]. pcDNA-HBEGF and pcDNA-PSMB5 were cloned by replacing the KRAB-dCas9 element of pHR-SFFV-KRAB-dCas9-P2A-mCherry (Addgene) with the human HBEGF or PSMB5 coding sequence and 3×FLAG. Vectors expressing cDNA of HBEGF with single-a.a. deletions were constructed via PCR-based site-directed mutagenesis (PfuUltraII Fusion HS DNA Polymerase, STRATAGENE). The primers used for these purposes are listed in Additional file 5: Table S7. Vectors expressing cDNAs of PSMB5 M104 and V90 substitutions were constructed via PCR-based site-directed mutagenesis (PrimeSTAR HS DNA Polymerase, Takara). Primers used for the construction of M104 and V90 substitution mutants are listed in Additional file 5: Table S10.

### sgRNA library design

The hg19 CDS sequences of targeted genes were downloaded from UCSC genome browser (https://genome.ucsc.edu/), and all potential sgRNAs with NAG or NGG PAM sequence were designed using a homemade script to build the library.

### Construction of the CRISPR/Cas9 sgRNA library

Two tiling libraries were constructed to include 1236 and 3712 sgRNAs targeting 3 drug-associated proteins and 3 toxin receptors, respectively (Additional file 4: Table S3). Array-based oligos encoding sgRNAs were synthesized and amplified via PCR with corresponding primers (Additional file 5: Table S4) that included the BsmBI recognition site at the 5′ end. The amplified DNA products were ligated into the vector using the Golden Gate method. The ligation mixture was then transformed into Trans1-T1 competent cells (Transgen) to generate the plasmid library [23, 29, 46]. The sgRNA plasmid library was subsequently transfected into HEK293T cells, together with two viral packaging plasmids, pVSVG and pR8.74 (Addgene), using the X-tremeGENE HP DNA transfection reagent (Roche). HeLa cells were then infected with a low MOI (~ 0.3) of lentivirus, and EGFP^+^ cells were collected 48 h after infection via FACS.

### Genome preparation and sequencing

Genomic DNAs of single site mutagenesis of HBEGF and CSPG4 were extracted after culturing for 14 days using the DNeasy Blood and Tissue kit (Qiagen). The targeted regions were amplified via 24 cycles of PCR (NEBNext Ultra II Q5 Master Mix). For each targeted region, 10 different forward primers and 10 different reverse primers were used to increase the diversity of the NGS library, each of which contained 1–10 additional nucleotides [47] (Additional file 2: Table S1). So, the potential bias of Illumina Sequencing that may affect screening results could be minimized [47]. PCR products from each library were purified using the DNA Clean & Concentrator-5 kit (Zymo Research Corporation) and indexed with different adaptors (NEB #7335, #7500) for NGS analysis.

### Library screening

For TcdB screening, four 150-mm dishes were plated with 3.5 × 10^6^ cells each as one experimental replicate. For each round of screening, cells were treated with an appropriate concentration: 70 ng/ml for the first round and 100 ng/ml for the second and third rounds. The details of DT and PA/LFnDTA screenings were the same as described in our previous report [8]. For 6-TG screening, six 150-nm dishes were plated with 3 × 10^6^ cells for each experimental replicate. Two hundred fifty nanograms per milliliter of 6-TG was used in the first and second rounds, and 300 ng/ml 6-TG was used in the third round of screening. For Bortezomib screening, seven 150-nm dishes were plated with 2 × 10^6^ cells for each experimental replicate. For each round of screening, cells were treated with variable doses of Bortezomib as follows: 10 ng/ml for the first round, 16 ng/ml for the second round, 28 ng/ml for the third round, and 40 ng/ml for the fourth round. For BI2536 screening, two 150-nm dishes were plated with 3.5 × 10^6^ cells for each experimental replicate. For each round of screening, cells were treated with 4 ng/ml of BI2536 for the first round, 5 ng/ml for the second round, and 6 ng/ml for the third round.

The resistant cells from each screening were collected for genomic DNA and total RNA extraction, followed by reverse transcription. The sgRNA-coding regions and cDNAs of the targeted genes obtained through PCR amplification were then subjected to NGS analysis.

### Identification of sgRNA sequences

Genomic DNAs were extracted from library cells (cell number corresponding to 1000× sgRNA coverage) using DNeasy Blood and Tissue kit (Qiagen). sgRNA regions were amplified via 26 cycles of PCR using primers [5–8] annealing to the flanking sequences of the sgRNAs. PCR products from each replicate were purified with DNA Clean & Concentrator-5 (Zymo Research Corporation), indexed with different adaptors (NEB #7370, #7335, #7500), and analyzed via NGS.

### cDNA preparation and sequencing

Total RNAs were extracted from library cells using RNAprep Pure Cell/Bacteria Kit (TIANGEN), and cDNAs were synthesized using Quantscript RT Kit (TIANGEN). A two-step method was employed to construct libraries for NGS. The first step consisted of PCR amplification of the cDNA (26 cycles; PrimeSTAR HS DNA Polymerase, Takara). Primers used for different genes are listed in Additional file 5: Table S5. The coding sequence of *CSPG4* was approximately 6.9 kb in length, and three amplification reactions were employed to obtain overlapping fragments (~ 50 bp) encompassing its full length. After purified, cDNAs from each gene were sheared to ~ 250 bp using the Covaris S2 system (Additional file 1: Figure S3). The sheared products were purified and concentrated using DNA Clean & Concentrator-5 kit (Zymo Research Corporation) and indexed with different adaptors (NEB #7370, #7335, #7500) for NGS analysis.

### Evaluation of mutation variety generated by CRISPR mutagenesis

Sequencing reads were trimmed, and the remaining reads were filtered to remove those with base quality below 30 before subjected to mapping with the reference sequences of targeted genes using Bowtie2 2.3.4.3 and sorted using SAMtools 1.9. Because different samples had variable volumes of sequencing data, mapped reads were down sampled (by sambamba-0.6.9) to approximately 100,000 reads for further analysis. Mutation types of both sgRNA libraries and wild type controls (without sgRNA) were calculated (using R package “CrispRVariants”) covering ± 20 nt of estimated Cas9 cutting sites (3-bp upstream of PAM). Mutations with read counts less than 5 were removed from the analysis.

### Computational methods for the identification of critical amino acids

Sequencing reads were trimmed, filtered, and mapped as described above. We only considered those reads containing in-frame mutations leading to either a.a. deletion, combo mutation, or a.a. substitution. Here, mutations with read count < 9 were also removed.

For fragments leading to a.a. deletions, we computed the deletion frequency (Freq^del^) for each deletion type. For a deletion type *x*, we computed Freq^del _ *x*^ as follows:

$$ {\mathrm{Freq}}^{\mathrm{del}\_x}=\frac{\mathrm{number}\ \mathrm{of}\ \mathrm{reads}\ \mathrm{with}\ \mathrm{deletions}\ \mathrm{of}\ \mathrm{del}\_x}{\mathrm{total}\ \mathrm{number}\ \mathrm{of}\ \mathrm{reads}\ \mathrm{covered}\ \mathrm{region}\ \mathrm{del}\_x} $$

For fragments containing a.a. combo mutations, their frequencies (Freq^combo^) were calculated. For a combo mutation type *y*, we calculated Freq^combo _ *y*^ as follows:

$$ {\mathrm{Freq}}^{\mathrm{combo}\_y}=\frac{\mathrm{number}\ \mathrm{of}\ \mathrm{reads}\ \mathrm{with}\ \mathrm{combo}\ \mathrm{mutations}\ \mathrm{of}\ \mathrm{combo}\_y}{\mathrm{total}\ \mathrm{number}\ \mathrm{of}\ \mathrm{reads}\ \mathrm{covered}\ \mathrm{region}\ \mathrm{combo}\_y\ } $$

Then, fold change of a.a. deletion (*dfc*) and a.a. combo mutation (*cmfc*) were calculated, respectively. For a deletion type *x* and combo mutation type *y*, *dfc* _ *x* and *cmfc* _ *y* were calculated as follows:

$$ dfc\_x=\frac{{\mathrm{Freq}}^{\mathrm{del}\_x}\ \mathrm{after}\ \mathrm{screening}}{{\mathrm{Freq}}^{\mathrm{del}\_x}\ \mathrm{before}\ \mathrm{screening}} $$

$$ cmfc\_y=\frac{{\mathrm{Freq}}^{\mathrm{combo}\_y}\ \mathrm{after}\ \mathrm{screening}}{{\mathrm{Freq}}^{\mathrm{combo}\_y}\ \mathrm{before}\ \mathrm{screening}} $$

To estimate the significance of individual amino acid, length of affected a.a. and its position (*dfc* and *cmfc*) were taken into calculation. Fold change of single-a.a. deletion was assigned a weight (*w*). Fold changes of multiple-a.a. deletion and combo mutation (*dfc* and *cmfc*) were divided by squared of affected length, and the value was assigned to each affected amino acid.

That is,

$$ { df c}_{\mathrm{a}.\mathrm{a}{.}_i}=w\times dfc\ \mathrm{of}\ \mathrm{single}\ \mathrm{a}.\mathrm{a}{.}_i+{\sum}_j\frac{df{c}_j\ \mathrm{a}\mathrm{ffect}\ \mathrm{a}.\mathrm{a}{.}_i\ }{{\left(\mathrm{length}\ \mathrm{of}\  df{c}_j\right)}^2} $$

$$ { cmf c}_{\mathrm{a}.\mathrm{a}{.}_i}={\sum}_k\frac{cmf{c}_k\ \mathrm{a}\mathrm{ffect}\ \mathrm{a}.\mathrm{a}{.}_i\ }{{\left(\mathrm{length}\ \mathrm{of}\  cmf{c}_k\right)}^2} $$

Where, a. a._*i*_ is the *i*th amino acid along with the targeted protein, *dfc*_*j*_ is the fold change of *j*th a.a. deletion which affect a. a._*i*_, and *cmfc*_*k*_ is the fold change of *k*th a.a. combo mutation which affect a. a._*i*_.

For fragments containing a.a. substitutions, we computed the substitution ratio of amino acid *i* (${\mathrm{Freq}}_{\mathrm{Aa}.\mathrm{a}{.}_i}^{\mathrm{sub}}$) as follows:

$$ {\mathrm{Freq}}_{\mathrm{a}.\mathrm{a}{.}_i}^{\mathrm{sub}}=\frac{\mathrm{number}\ \mathrm{of}\ \mathrm{reads}\ \mathrm{with}\ \mathrm{subsitutions}\ \mathrm{of}\ \mathrm{a}.\mathrm{a}{.}_i\ }{\mathrm{total}\ \mathrm{number}\ \mathrm{of}\ \mathrm{reads}\ \mathrm{covered}\ \mathrm{a}.\mathrm{a}{.}_i} $$

Because we could not quantify a.a. substitution frequency before the screening (Additional file 1: Figure S4), we estimated the effects of a.a. substitutions qualitatively by setting a cutoff frequency as follows:

$$ \mathrm{Cut}-{\mathrm{off}}^{\mathrm{sub}}=\mathrm{mean}\ \mathrm{of}\ \log 10\ {\mathrm{Freq}}^{\mathrm{sub}}+3\times \mathrm{standard}\ \mathrm{deviation}\ \mathrm{of}\ \log 10\ {\mathrm{Freq}}^{\mathrm{sub}} $$

We gave a qualitative score to amino acid substitution frequency (sf_score) as follows:

$$ \mathrm{sf}\_{\mathrm{score}}_{\mathrm{a}.\mathrm{a}{.}_i}=\left\{\begin{array}{c}2,{\mathrm{Freq}}_{\mathrm{a}.\mathrm{a}{.}_i}^{\mathrm{sub}}>{\mathrm{Cutoff}}^{\mathrm{sub}}\\ {}0,{\mathrm{Freq}}_{\mathrm{a}.\mathrm{a}{.}_i}^{\mathrm{sub}}\le {\mathrm{Cutoff}}^{\mathrm{sub}}\end{array}\right) $$

Finally, we estimated the functional importance of each amino acid in a semi-quantitative way by assigning essential scores:

$$ \mathrm{quantitative}\_{\mathrm{effect}}_{\mathrm{a}.\mathrm{a}{.}_i}=\mathrm{normalization}\ \mathrm{of}\ \log \left({dfc}_{\mathrm{a}.\mathrm{a}{.}_i}+{cmfc}_{\mathrm{a}.\mathrm{a}{.}_i}\right) $$

$$ {\mathrm{Score}}_{\mathrm{a}.\mathrm{a}{.}_i}=-\log (p)\mathrm{of}\ \mathrm{quantitative}\_{\mathrm{effect}}_{\mathrm{a}.\mathrm{a}{.}_i}+\mathrm{sf}\_{\mathrm{score}}_{\mathrm{a}.\mathrm{a}{.}_i} $$

### Validation of the screening results

For the validation of critical substitutions of PSMB5 and PLK1, sgRNAs were designed near the mutation sites, and each 119-nt ssODN donor encoded one amino acid substitution for a validated residue. All sgRNAs and ssODN donor sequences are listed in Additional file 5: Table S8. HeLa cells were transfected with 1 μg of sgRNA and 2 μg of the ssODN donor in six-well plates. Fourteen days post-transfection, 1.5 × 10^5^ cells were seeded in six-well plates 24 h before drug selection. Cells were treated with corresponding drugs at the proper dosages for 72 h: Bortezomib (10 ng/ml) and BI2536 (10 ng/ml). Genomic DNAs of drug-resistant cells were extracted using TIANamp Genomic DNA Kit (TIANGEN). The mutated loci were amplified using TransTaq DNA Polymerase High Fidelity (Transgen) and purified using a Universal DNA Purification Kit (TIANGEN). Primers are listed in Additional file 5: Table S9. PCR fragments were cloned into pEASY-T5 Zero Cloning Kit (Transgen) for sequencing.

### Western blotting

Cell lysates were resolved on 10% SDS/PAGE gels (Bio-rad) for electrophoresis and transferred to PVDF membrane (Millipore) by Trans-blot Turbo transfer system (Bio-rad). After blocking with 5% non-fat milk at 37 °C for 1 h, probed with anti-FLAG antibody (MBL) and anti-β-tubulin antibody (CWBIO) overnight at 4 °C, the membrane was incubated with goat anti-mouse IgG-HPR secondary antibody (Jackson Immunoresearch) 1 h at room temperature. Clarity Western ECL Substrate Kit (Bio-rad) and Chemi-doc system (Bio-rad) were used to detect protein bands.

### Cytotoxicity assay

Cells were seeded in 96-well plates 24 h before drug or toxin treatment (5000 cells for diphtheria toxin and 3000 cells for Bortezomib), and different concentrations of Bortezomib or DT were added. Cells were incubated at 37 °C for 48 h (DT) or 72 h (Bortezomib) before the addition of 1 mg/ml of MTT (3-[4,5-dimethylthiazol-2-yl]-2,5-diphenyltetrazolium bromide) [25, 48]. Spectrophotometer readings at 570 nm were collected using BioTek Cytation5 (BioTek Instruments).

### Structure analysis

Structures of the complex of diphtheria toxin with EGF-like domain of HBEGF (PDB code: 1XDT) [49], PSMB5 with Bortezomib (PDB code: 5LF3, 2F16, 4QVN, and 4QVQ) [50–52], PLK1 with BI2536 (PDB code: 2RKU) [42], and HPRT1 bound with GMP (PDB code: 1HMP) [53] were downloaded from the Protein Data Bank. The structures were analyzed using the PyMOL Molecular Graphics System, version 2.0, Schrödinger, LLC (https://pymol.org/2/).

## Supplementary information

**Additional file 1: Figure S1.** Rationale of acquiring residues critical for protein function based on phenotypic changes associated with in-frame mutations (copy number of targeted gene: *n* = 2). **Figure S2.** CRISPR mediated single site mutagenesis of *HBEGF* and *CSPG4*. **Figure S3.** Experimental conditions for PASTMUS screening. **Figure S4.** Distribution of amino acid (a.a.) deletion (a), combo mutation (b) and a.a. substitution (c) frequency in the original libraries (before screening) and the mock controls (wild type without sgRNA targeting). **Figure S5.** Relation between mutation affected length and a.a. coverage in libraries before screening. **Figure S6.** Scatter plot of sgRNA fold changes after screening on a log scale between two replicates. **Figure S7.** Expression of each HBEGF mutant for validation. **Figure S8.** Identification of ANTXR1 amino acids critical for PA/LFnDTA mediated cytotoxicity through PASTMUS. **Figure S9.** Identification of CSPG4 amino acids critical for TcdB mediated cytotoxicity through PASTMUS. **Figure S10.** Identification of HPRT1 amino acids critical for 6-TG mediated killing through PASTMUS. **Figure S11.** Identification of PLK1 amino acids critical for BI2536 mediated killing through PASTMUS. **Figure S12.** Sequencing chromatogram of mutated sites in PSMB5 locus from cells with or without ssODN donor transfection. **Figure S13.** DNA sequencing analysis of mutated alleles in the human PSMB5 locus from Bortezomib-resistant cell clones. **Figure S14.** Effects of PSMB5 V90E substitution variant on Bortezomib mediated killing. **Figure S15.** Correlation of NGS depth and data quality for PASTMUS screening of HBEGF. **Figure S16.** Correlation of NGS depth and data quality for PASTMUS of PSMB5.

**Additional file 2: Table S1**. Information of CRISPR mediated single site mutagenesis of *HBEGF* and *CSPG4*.

**Additional file 3: Table S2.** Information of six genes used in PASTMUS.

**Additional file 4: Table S3.** sgRNA sequences used for PASTMUS.

**Additional file 5: Table S4.** Primers used for sgRNA oligos amplification. **Table S5.** Primers used for cDNA amplification. **Table S7.** Primers used to generate different deletion mutants for *HBEGF*. **Table S8.** sgRNAs and ssODNs used for *PSMB5* and *PLK1* mutants validation. **Table S9.** Primers used for *PSMB5* genome amplification. **Table S10.** Primers used to generate PSMB5 M104 and V90 mutants. **Table S11.** Summary of candidate validations.

**Additional file 6: Table S6.** Essential scores of amino acids from six proteins through PASTMUS.

**Additional file 7:** **Video S1.** Crystal structure of the complex of DT toxin with EGF-like domain of HBEGF.

**Additional file 8: Video S2.** Crystal structure of PSMB5 with Bortezomib.

**Additional file 9: Video S3.** Crystal structure of PLK1 with BI2536.

**Additional file 10: Video S4.** Crystal structure of HPRT1 with bound GMP.

## Data Availability

The accession number for the raw sequencing data reported in this paper is NCBI Sequence Read Archive (SRA): SRP230665 (PRJNA590617) [54]. Source code written by R for PASTMUS is available at https://bitbucket.org/WeiLab/pastmus [55] and a demo of the computational pipeline at https://figshare.com/articles/PASTMUS_mapping_functional_elements_at_single_amino_acid_resolution_in_human_cells/10435370.
