# Supplementary material for: PASTMUS: mapping functional elements at single amino acid resolution in human cells
Source: Genome Biol. 2019 Dec 16;20:279. doi: 10.1186/s13059-019-1897-7 (PMC6913009; doi:10.1186/s13059-019-1897-7)
Supplement: Supplementary file 1 — Additional file 1: Figure S1. Rationale of acquiring residues critical for protein function based on phenotypic changes associated with in-frame mutations (copy number of targeted gene: n = 2). Figure S2. CRISPR mediated single site mutagenesis of HBEGF and CSPG4. Figure S3. Experimental conditions for PASTMUS screening. Figure S4. Distribution of amino acid (a.a.) deletion (a), combo mutation (b) and a.a. substitution (c) frequency in the original libraries (before screening) and the mock controls (wild type without sgRNA targeting). Figure S5. Relation between mutation affected length and a.a. coverage in libraries before screening. Figure S6. Scatter plot of sgRNA fold changes after screening on a log scale between two replicates. Figure S7. Expression of each HBEGF mutant for validation. Figure S8. Identification of ANTXR1 amino acids critical for PA/LFnDTA mediated cytotoxicity through PASTMUS. Figure S9. Identification of CSPG4 amino acids critical for TcdB mediated cytotoxicity through PASTMUS. Figure S10. Identification of HPRT1 amino acids critical for 6-TG mediated killing through PASTMUS. Figure S11. Identification of PLK1 amino acids critical for BI2536 mediated killing through PASTMUS. Figure S12. Sequencing chromatogram of mutated sites in PSMB5 locus from cells with or without ssODN donor transfection. Figure S13. DNA sequencing analysis of mutated alleles in the human PSMB5 locus from Bortezomib-resistant cell clones. Figure S14. Effects of PSMB5 V90E substitution variant on Bortezomib mediated killing. Figure S15. Correlation of NGS depth and data quality for PASTMUS screening of HBEGF. Figure S16. Correlation of NGS depth and data quality for PASTMUS of PSMB5. [file 13059_2019_1897_MOESM1_ESM.pdf]

Mapping functional elements at single amino acid resolution in human cells

Xinyi Zhang, Di Yue, Yinan Wang, Yuexin Zhou, Ying Liu, Yeting Qiu, Feng Tian,

Ying Yu, Zhuo Zhou, Wensheng Wei

Biomedical Pioneering Innovation Center (BIOPIC), Beijing Advanced Innovation Center for Genomics, Peking-Tsinghua Center for Life Sciences, Peking University Genome Editing Research Center, State Key Laboratory of Protein and Plant Gene Research, School of Life Sciences, Peking University, Beijing 100871, China.

Academy for Advanced Interdisciplinary Studies, Peking University, Beijing 100871, China.

Correspondence should be addressed to W.W. (wswei@pku.edu.cn).

Copy number of targeted gene: n = 2			Loss-of-function mutant (recessive)		Gain-of-function mutant (dominant)		
**Genotype**	**Protein Expression**	**Protein Function**	**Loss-of-function**	**Enriched from Screening**	**Gain-of-function**	**Enriched from Screening**	**Note**
		+	No	No	No	No	
		+	No	No	No	No	
		+	No	No	No	No	
		+	No	No	Yes	Yes	
		—	Yes	Yes only for non-essential gene	No	No	
		+	No	No	No	No	
		—	Yes	Yes	Yes	Yes	
		+	No	No	No	No	
		+	No	No	Yes	Yes	Source of false positive
		—	Yes	Yes	Yes	Yes	

Figure S1 Rationale of acquiring residues critical for protein function based on phenotypic changes associated with in-frame mutations (copy number of targeted gene: n = 2).

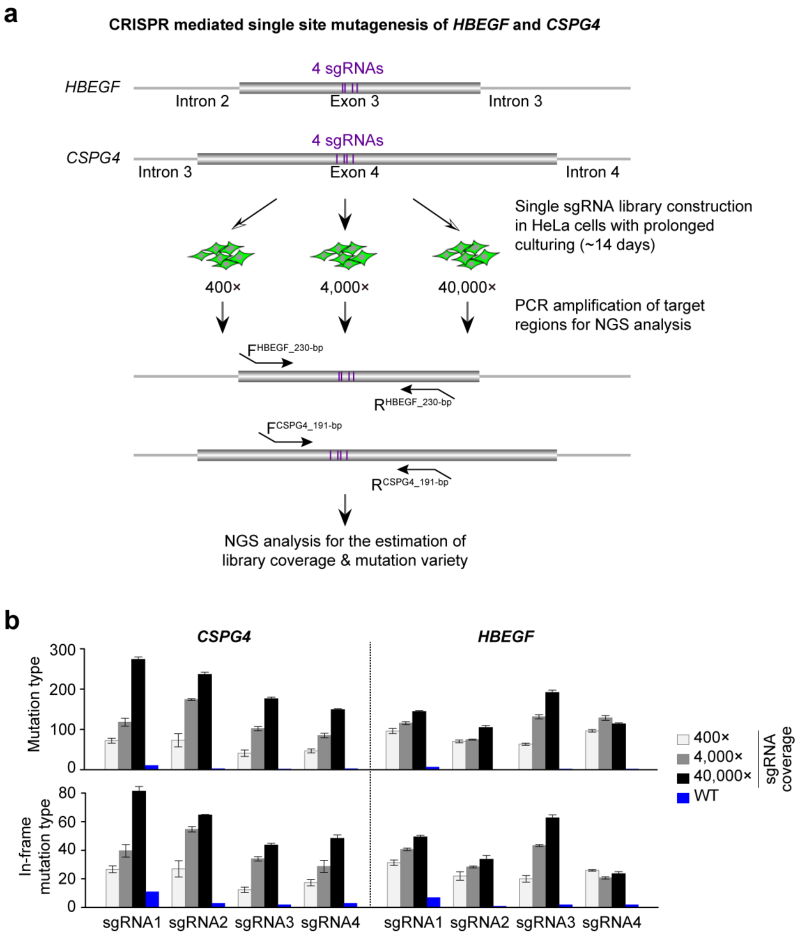

Figure S2 CRISPR mediated single site mutagenesis of *HBEGF* and *CSPG4*. **a** Workflow of CRISPR mutagenesis on eight sites from *HBEGF* and *CSPG4* genes. **b** Mutation type analysis of individual sgRNA targeting *HBEGF* and *CSPG4* genes. All detected number (through NGS of 50,000×) of total mutation types (top) and in-frame mutation types (bottom) with indicated sgRNA coverages (400×, 4,000×, 40,000×) and wild type control (without sgRNA targeting) are shown.

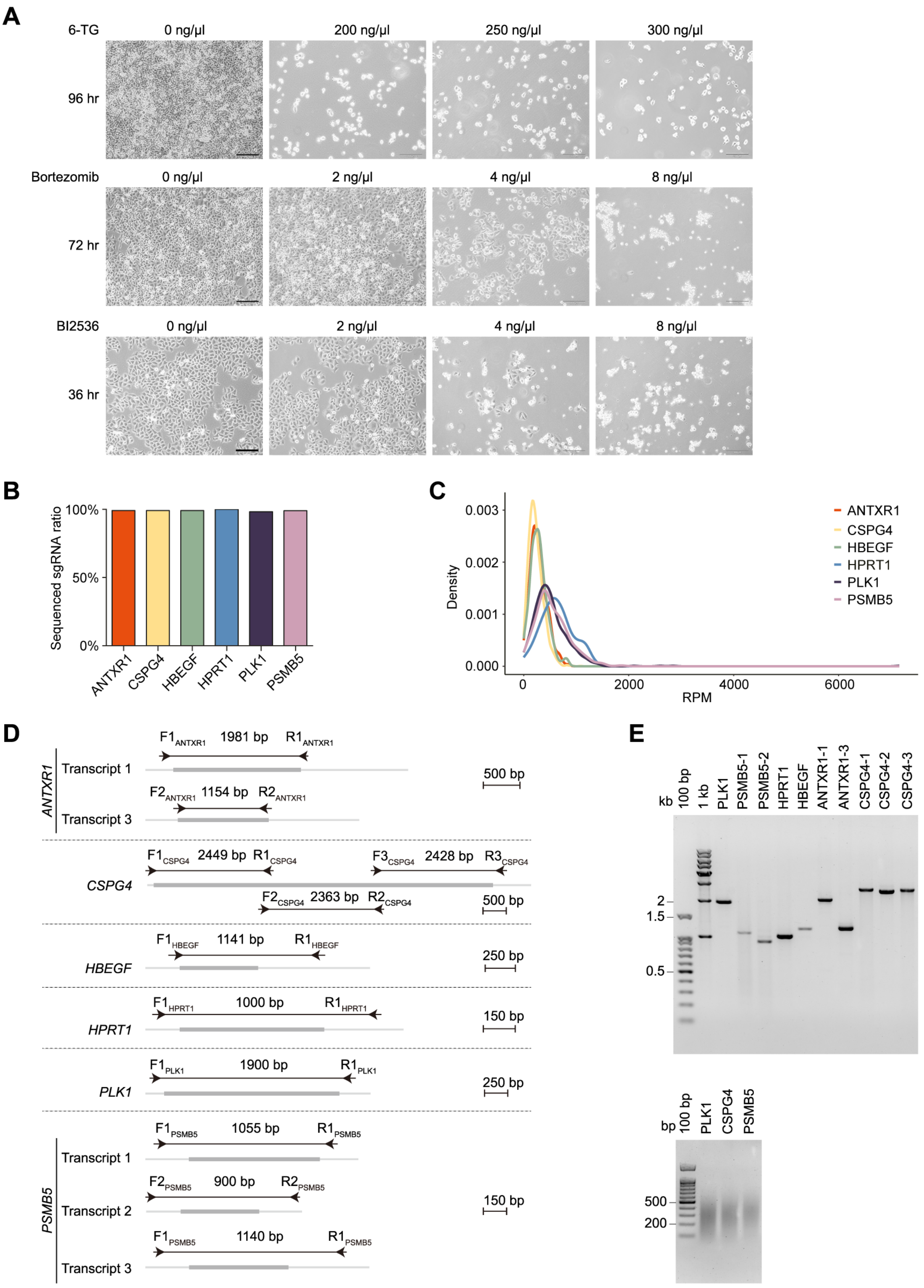

Figure S3 Experimental conditions for PASTMUS screening. a Dosage effects of three cancer drugs on HeLa cell killing for the indicated treatment times. **b** Recovery of sgRNAs in the libraries. **c** The relative read count distribution of each sgRNA in the libraries. **d** Schematic representation of PCR amplification of targeted cDNAs. The primers employed for different genes are listed in the Additional file 2: Table S5. **e** PCR amplification of

targeted cDNAs (top) and shearing of DNA fragments to an average length of 250-bp (bottom).

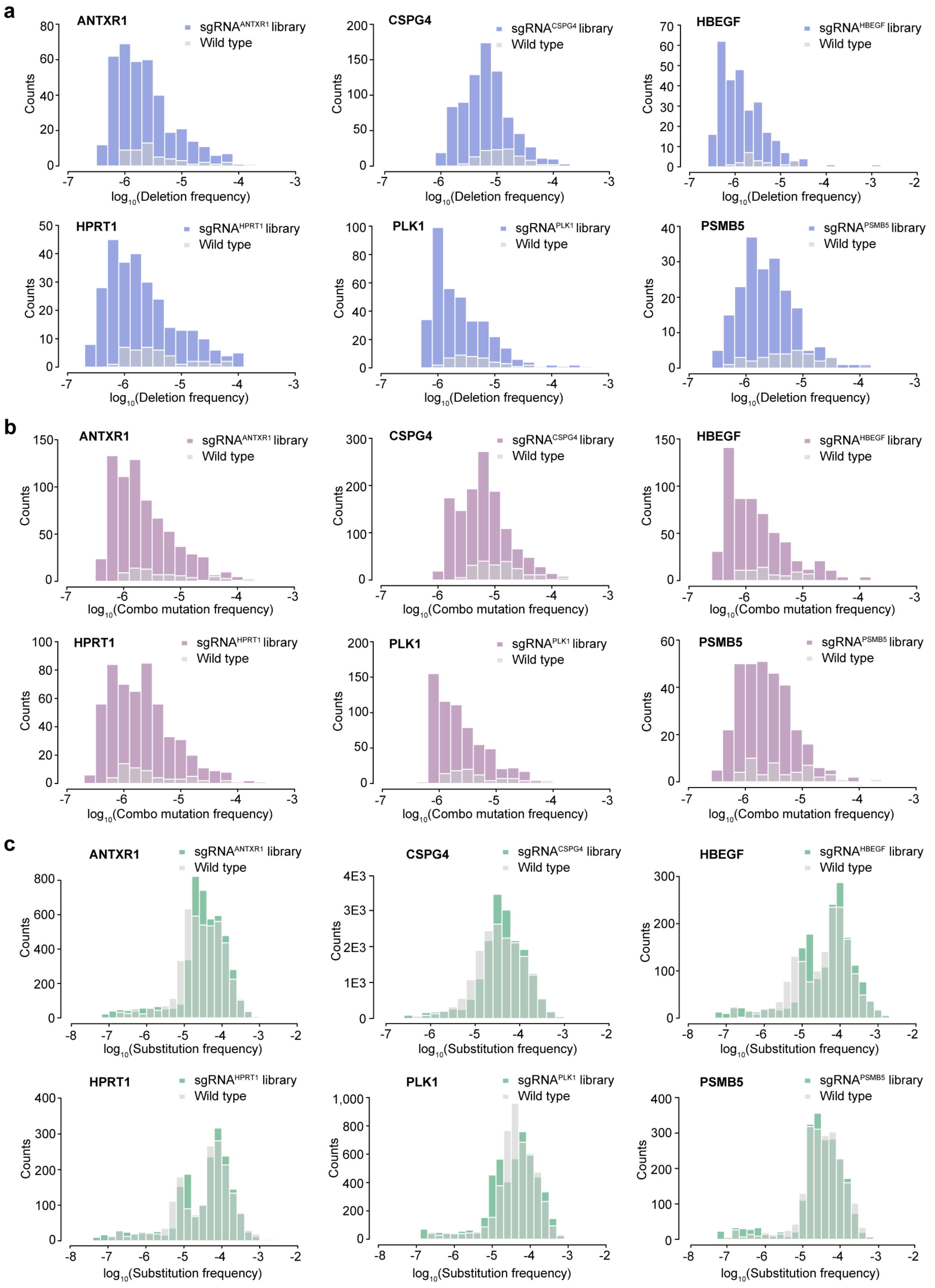

Figure S4 Distribution of amino acid (a.a.) deletion (a), combo mutation (b) and a.a. substitution (c) frequency in the original libraries (before screening) and the mock controls (wild type without sgRNA targeting).

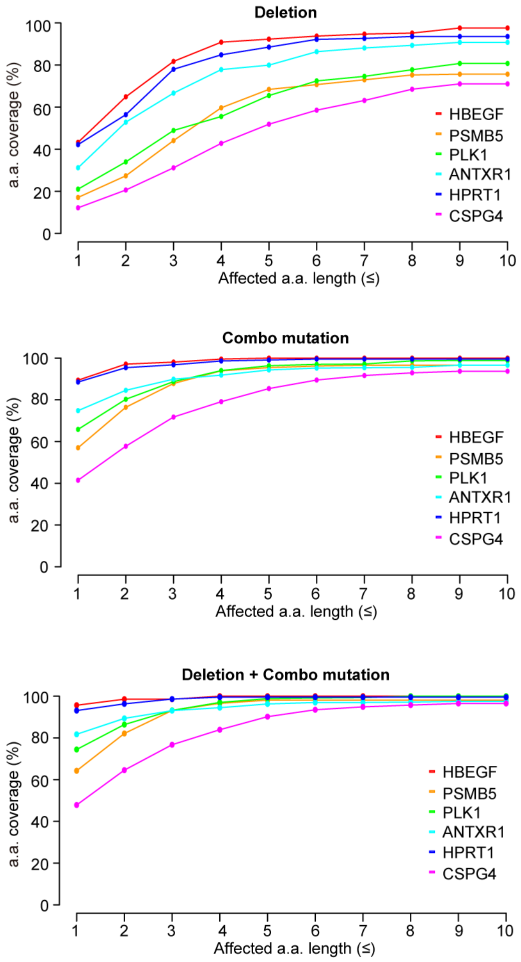

Figure S5 Relation between mutation affected length and a.a. coverage in libraries before screening. a.a. coverage is calculated within the indicated number of affected amino acids.

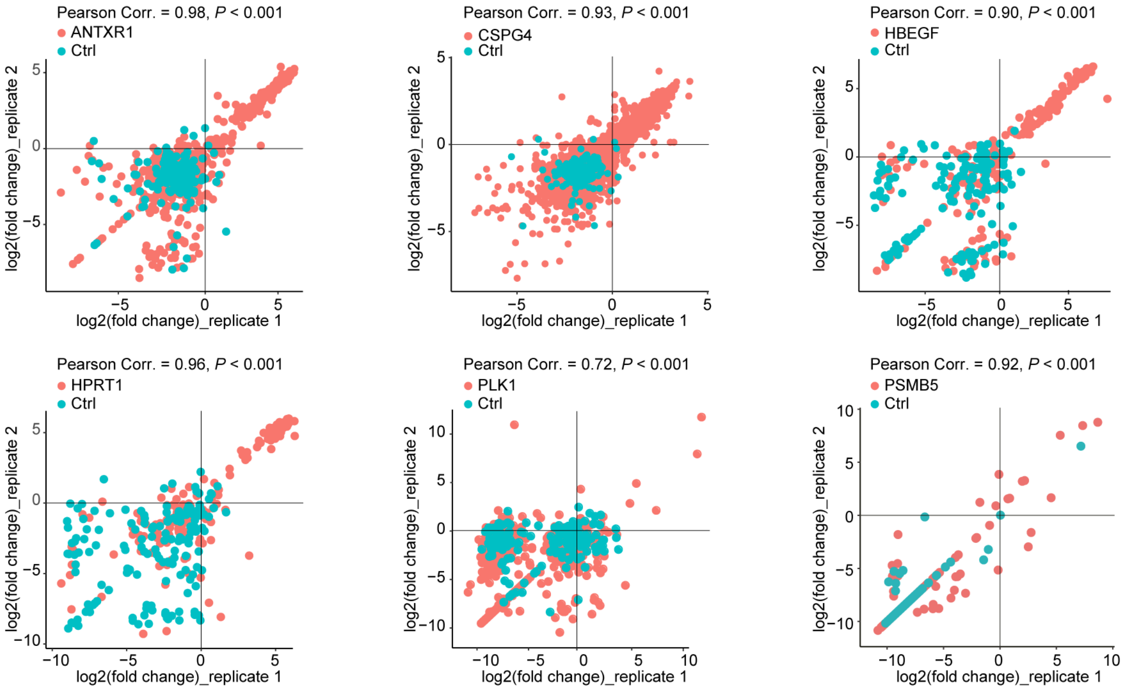

Figure S6 Scatter plot of sgRNA fold changes after screening on a log scale between two replicates.

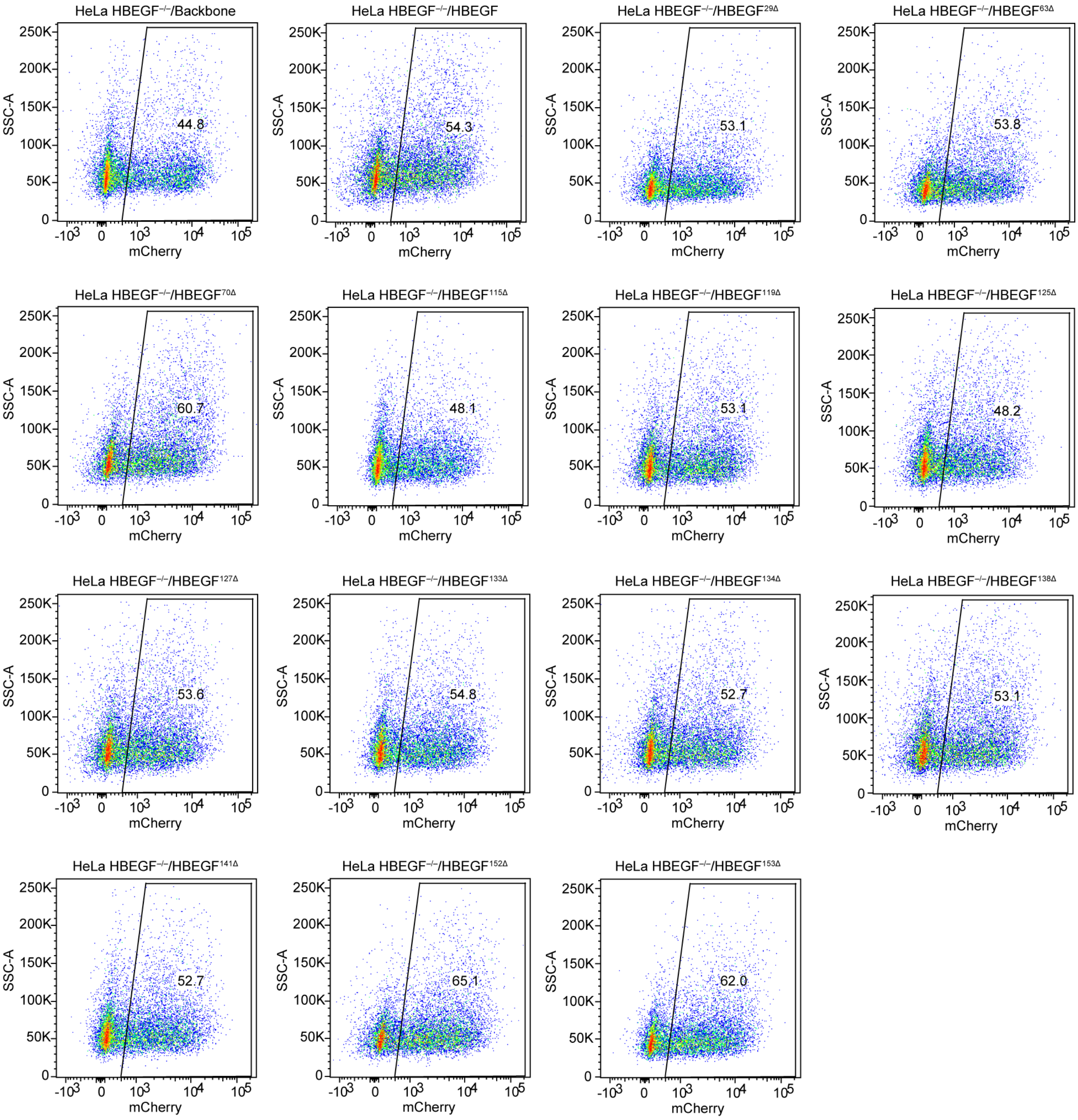

Figure S7 Expression of each HBEGF mutant for validation.

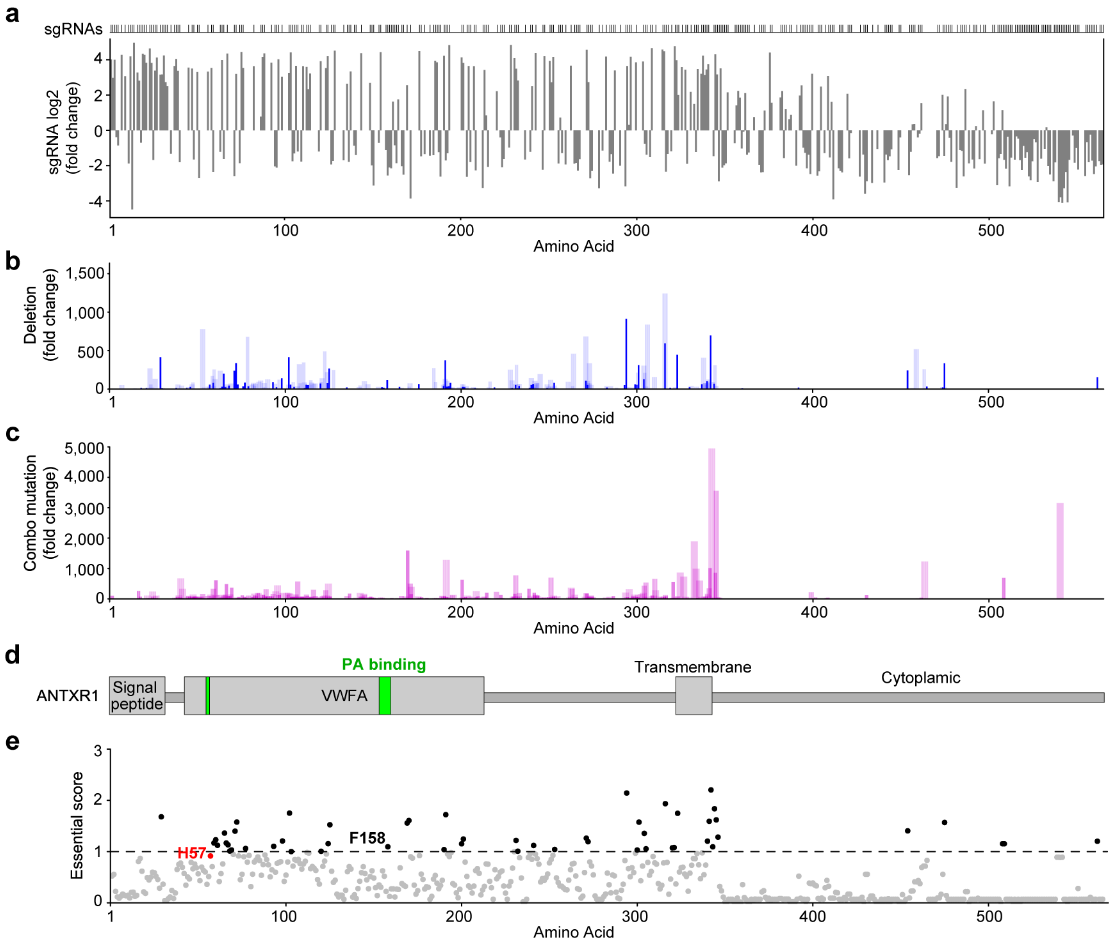

Figure S8 Identification of ANTXR1 amino acids critical for PA/LFnDTA mediated cytotoxicity through PASTMUS. **a** Identification of *ANTXR1*-targeting sgRNAs conferring cell resistance to PA/LFnDTA. Distribution of sgRNAs mapped to corresponding amino acid in ANTXR1 is indicated on top. **b** a.a. deletion fold change corresponding to each a.a. **c** a.a. combo mutation fold change corresponding to each a.a. **d** Schematic diagram of ANTXR1 domain with PA binding site shown in green. **e** Essential score of each a.a. of ANTXR1. Cut-off of essential score is plotted as a dash line, with critical amino acids above the cut-off shown in black and known critical amino acids labelled in red.

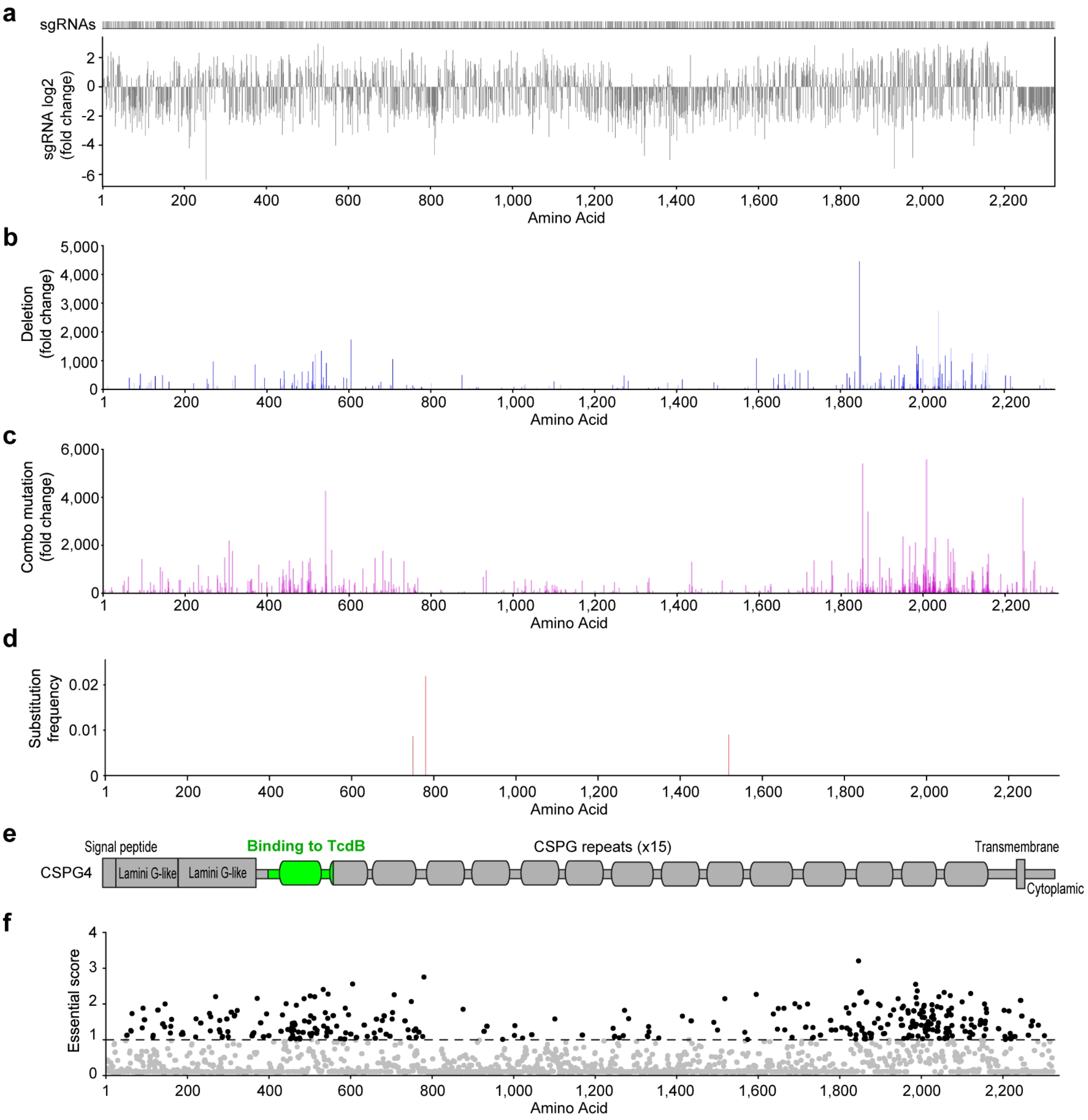

Figure S9 Identification of CSPG4 amino acids critical for TcdB mediated cytotoxicity through PASTMUS. **a** Identification of *CSPG4*-targeting sgRNAs conferring cell resistance to TcdB. Distribution of sgRNAs mapped to corresponding amino acid in CSPG4 is indicated on top. **b** a.a. deletion fold change corresponding to each a.a. **c** a.a. combo mutation fold change corresponding to each a.a. **d** a.a. substitution frequency corresponding to each a.a. **e** Schematic diagram of CSPG4 domain with TcdB binding site shown in green. **f** Essential score of each a.a. of CSPG4. Cut-off of essential score is plotted as a dash line, with critical amino acids above the cut-off shown in black.

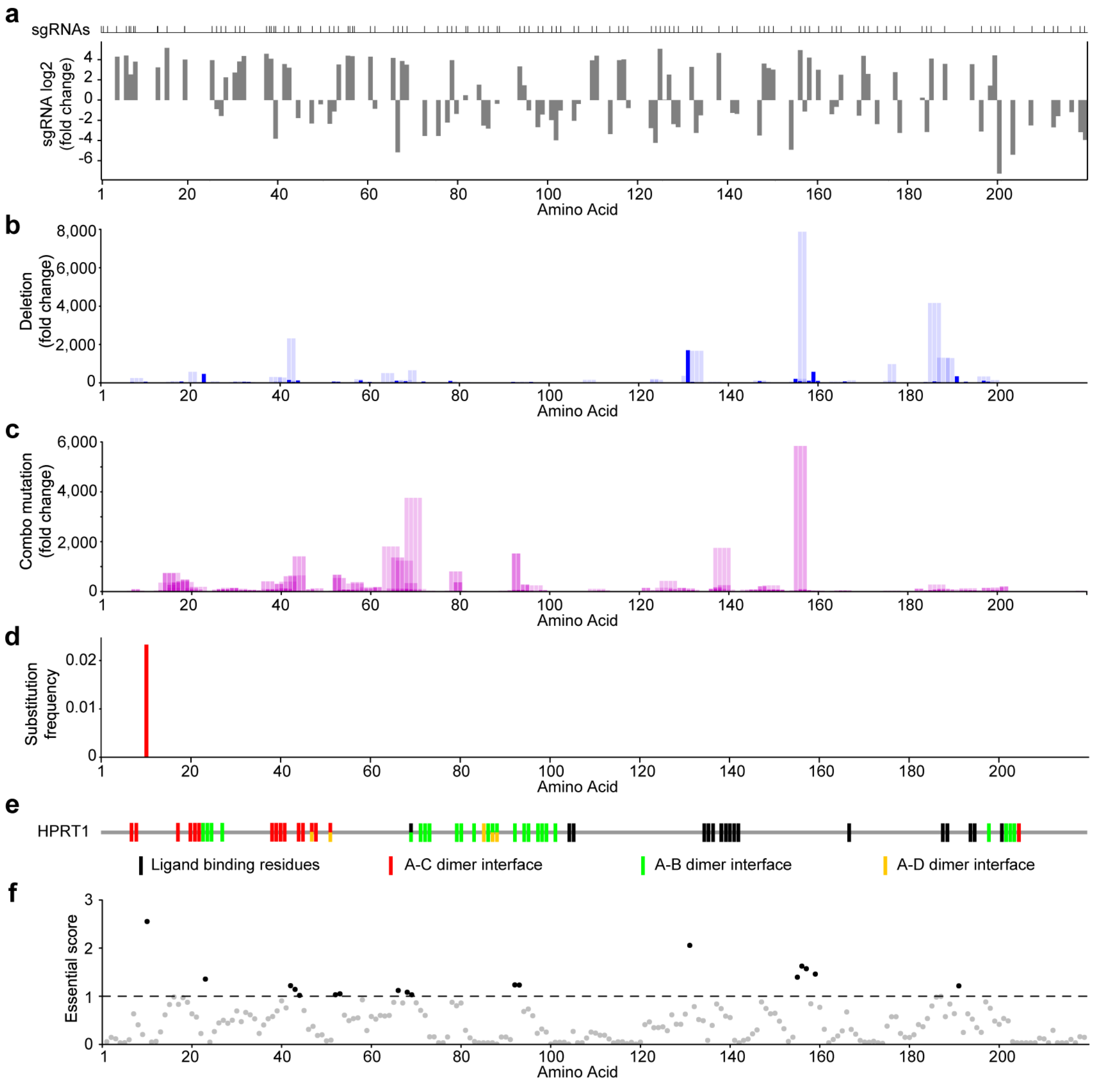

Figure S10 Identification of HPRT1 amino acids critical for 6-TG mediated killing through PASTMUS. **a** Identification of *HPRT1*-targeting sgRNAs conferring cell resistance to 6-TG. Distribution of sgRNAs mapped to corresponding amino acid in HPRT1 is indicated on top. **b** a.a. deletion fold change corresponding to each a.a. **c** a.a. combo mutation fold change corresponding to each a.a. **d** a.a. substitution frequency corresponding to each a.a. **e** Schematic diagram of HPRT1 domain with 6-TG binding site shown in green. **f** Essential score of each a.a. of HPRT1. Cut-off of essential score is plotted as a dash line, with critical amino acids above the cut-off shown in black.

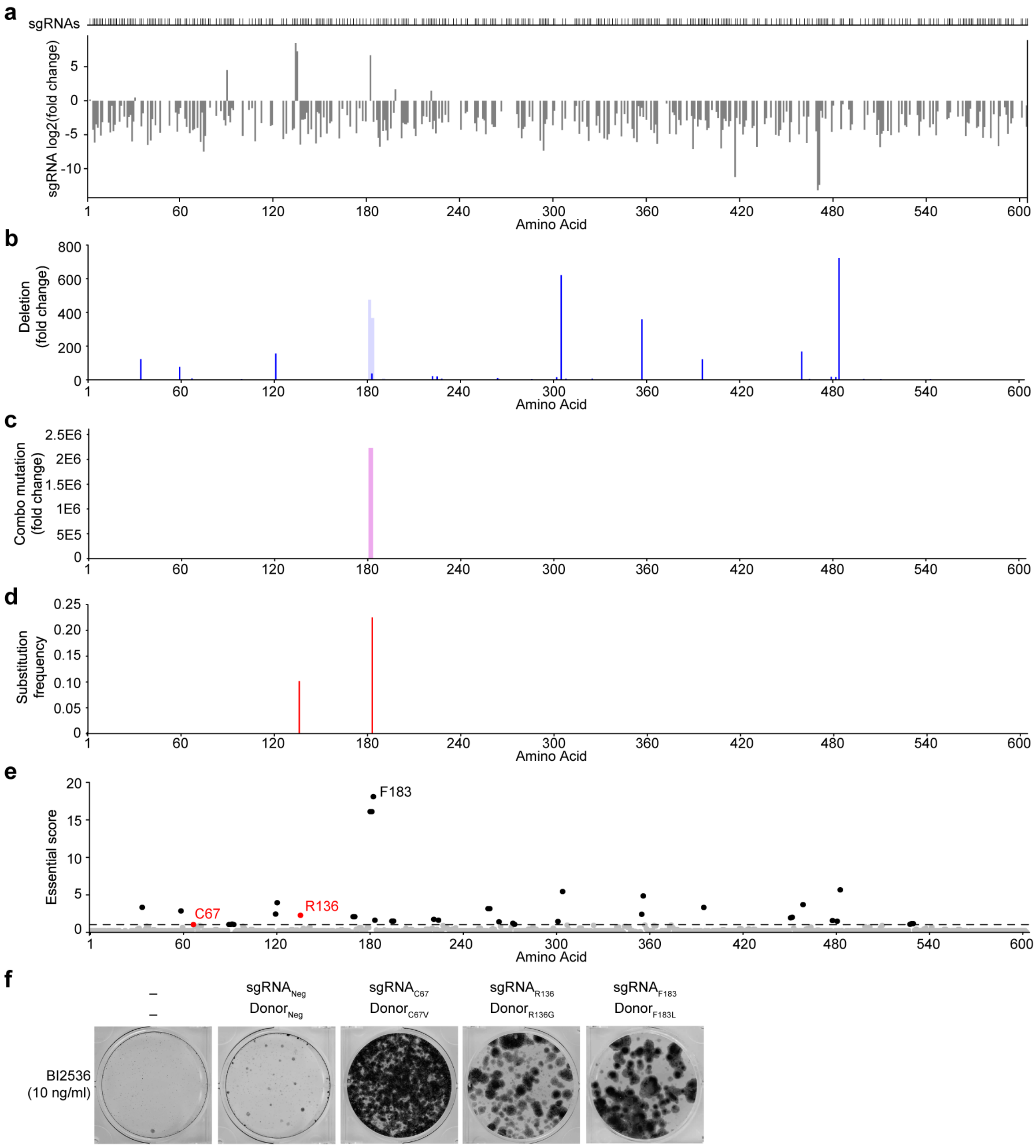

Figure S11 Identification of PLK1 amino acids critical for BI2536 mediated killing through PASTMUS. **a** Identification of *PLK1*-targeting sgRNAs conferring cell resistance to BI2536. Distribution of sgRNAs mapped to corresponding amino acid in PLK1 is indicated on top. **b** a.a. deletion fold change corresponding to each a.a. **c** a.a. combo mutation fold change corresponding to each a.a. **d** a.a. substitution frequency corresponding to each a.a. **e** Essential score of each a.a. of PLK1. Cut-off of essential score is plotted as a dash line, with critical amino acids above the cut-off shown in black and known critical amino acids labelled in red.

f MTT viability assay for the effects of the indicated substitutions of PLK1 on the susceptibility of cells to BI2536.

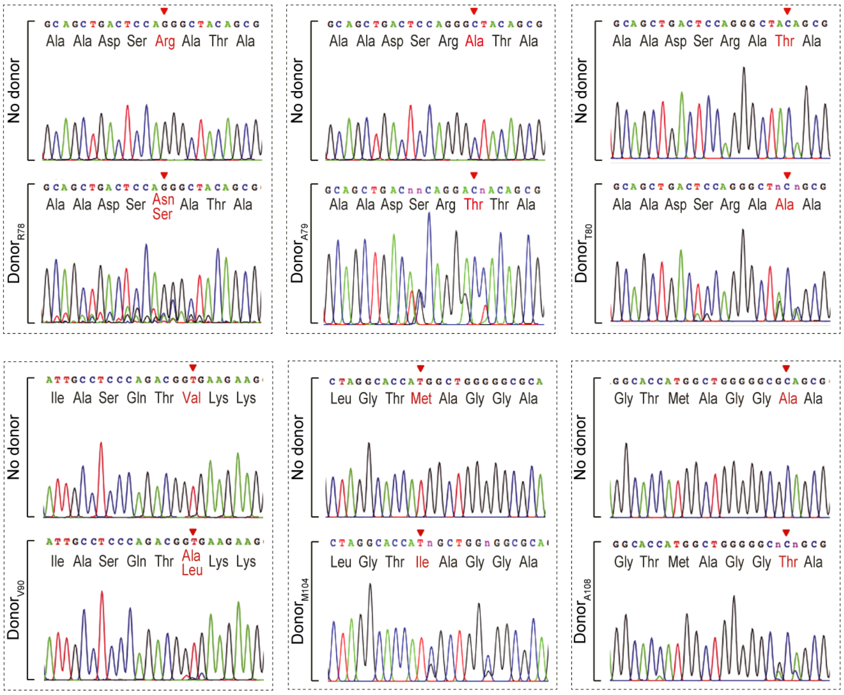

Figure S12 Sequencing chromatogram of mutated sites in *PSMB5* locus from cells with or without ssODN donor transfection. The mutated amino acids are shown in red.

HeLa PSMB5^{R78N} clone

	TGACTCCAGGGCTACAGCGGGTGCTTACATTGC	Wild type^{PSMB5}
PSMB5^{Mut}	TGACAGcAACGCTACAGCGGGTGCTTACATTGC	R78N^{PSMB5}, 4/13
	TGACTCCAGGGCTACAGCGGGTGCTTACATTGC	Wild type^{PSMB5}, 9/13

HeLa PSMB5^{V90L} clone 1

	GCCTCCCAGACGGTGAAGAAGGTGATAGAGATC	Wild type^{PSMB5}
PSMB5^{Mut}	GCCTCCCAGACGTTGAAGAAGGTGATAGAGATC	V90L^{PSMB5}, 4/8
	GCCTCCCAGACGGTGAAGAAGGTGATAGAGATC	Wild type^{PSMB5}, 4/8

HeLa PSMB5^{V90L} clone 2

	GCCTCCCAGACGGTGAAGAAGGTGATAGAGATC	Wild type^{PSMB5}
PSMB5^{Mut}	GCCTCCCAGACGTTGAAGAAGGTGATAGAGATC	V90L^{PSMB5}, 4/9
	GCCTCCCAGACGGTGAAGAAGGTGATAGAGATC	Wild type^{PSMB5}, 5/9

HeLa PSMB5^{A108T} clone

	AGGCACCATGGCTGGGGGCGCAGCGGATTGCAG	Wild type^{PSMB5}
PSMB5^{Mut}	AGGCACAATGGCTGGGGGCACCGCGGATTGCAG	A108T^{PSMB5}, 6/11
	AGGCACCATGGCTGGGGGCGCAGCGGATTGCAG	Wild type^{PSMB5}, 5/11

Figure S13 DNA sequencing analysis of mutated alleles in the human *PSMB5* locus from Bortezomib-resistant cell clones. sgRNA sequences are underlined; the shaded nucleotides represent the PAM sequences; red letters indicate wild type nucleotides and the tall letters indicate mutated nucleotides.

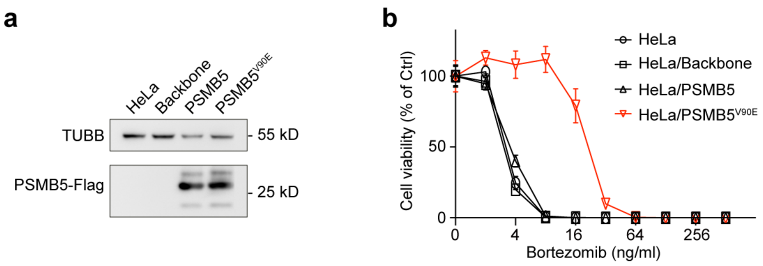

Figure S14 Effects of PSMB5 V90E substitution variant on Bortezomib mediated killing. **a** The expression of PSMB5 V90E substitution variants. **b** Effects of the PSMB5 V90E on the susceptibility of cells to Bortezomib. Data are presented as the mean ± s.d., n = 6.

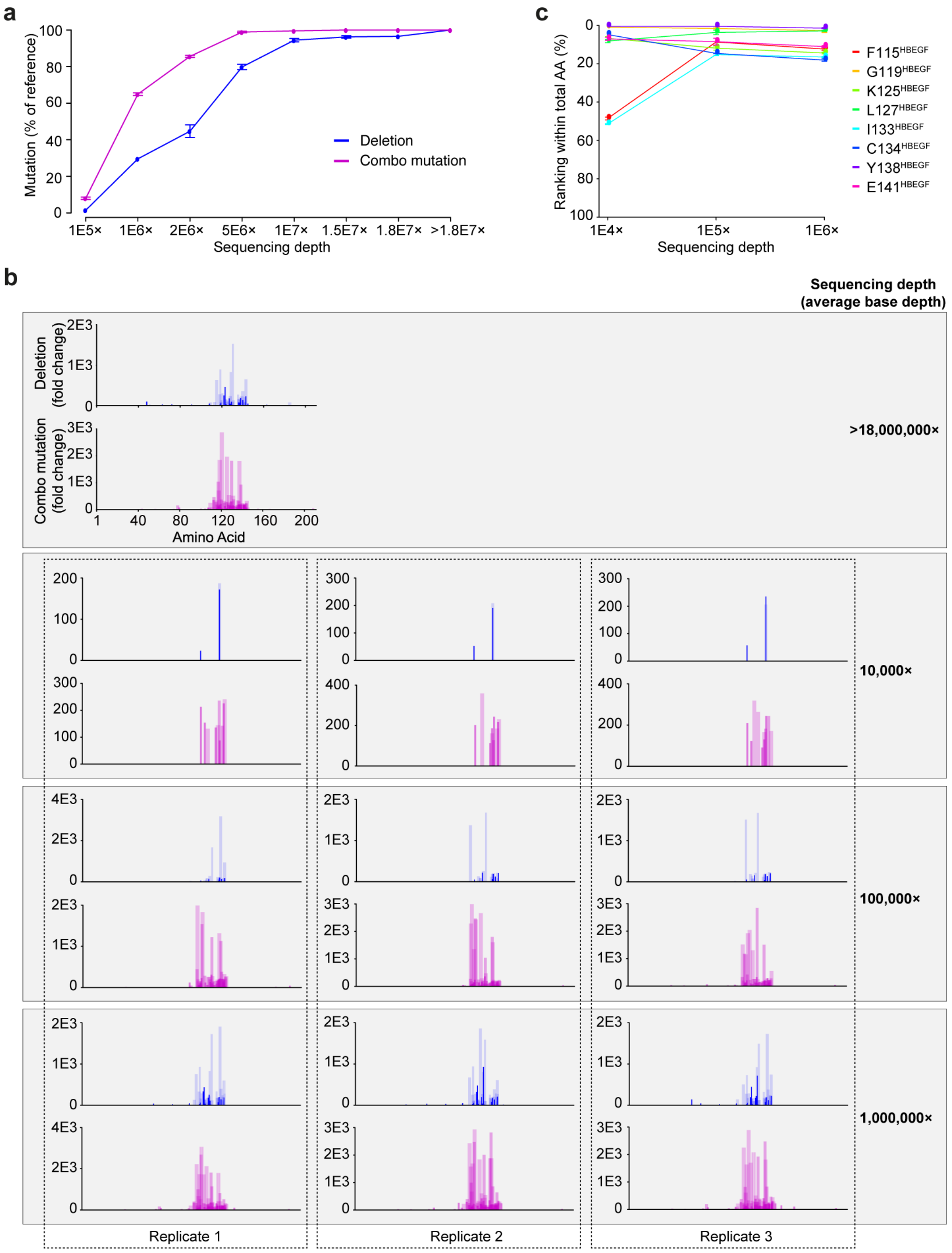

Figure S15 Correlation of NGS depth and data quality for PASTMUS screening of HBEGF. **a** Sequencing depth determination of HBEGF library before screening. **b** a.a. deletion and combo mutation fold changes corresponding to each a.a. at sequencing depths ranging from 1E4× to 1E6× of library after screening and 1.5E7× of library before screening. **c** Ranking of validated critical sites at corresponding sequencing depths.

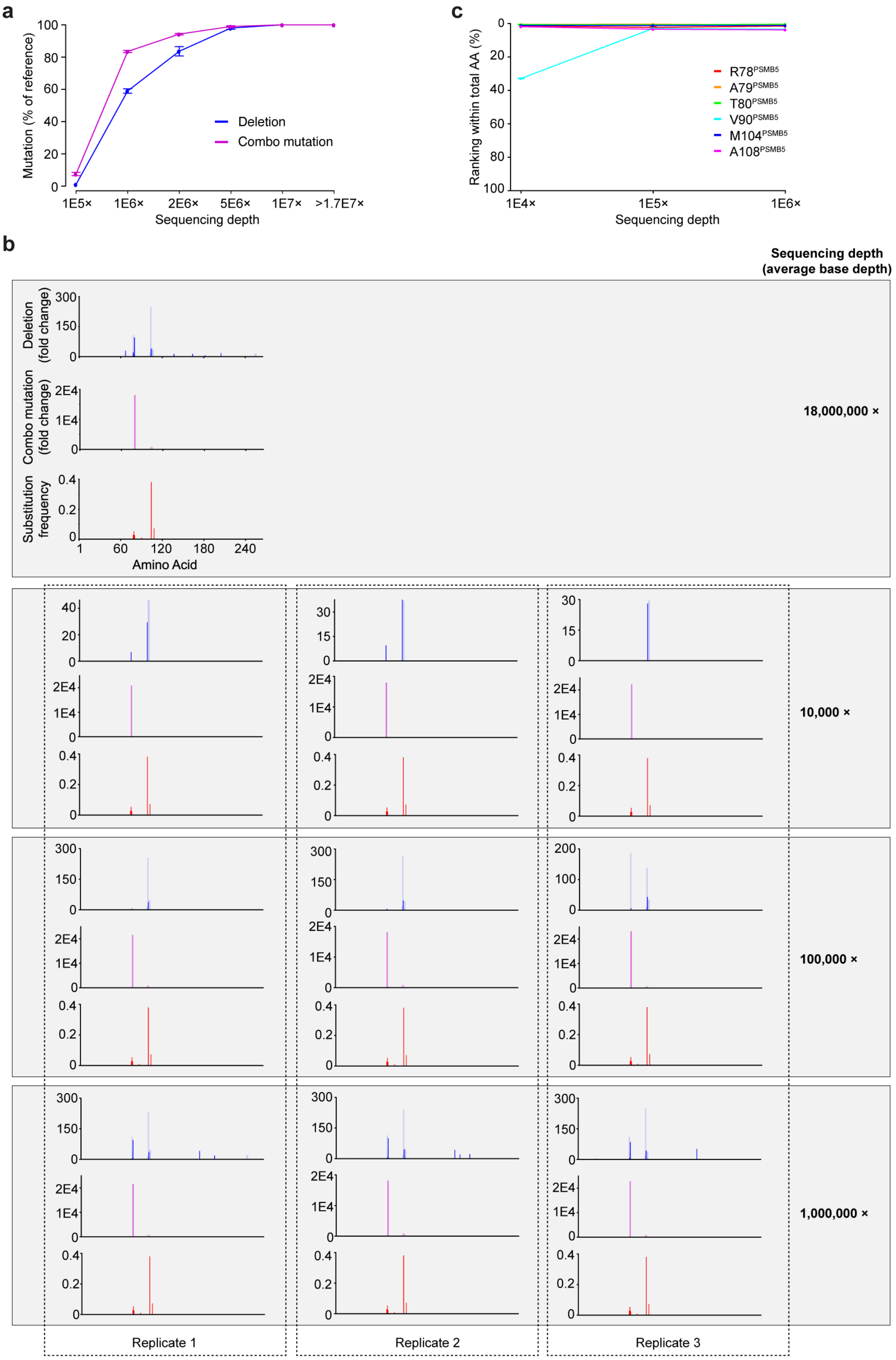
a
Mutation (% of reference)
100
80
60
40
20
0
1E5×
1E6×
2E6×
5E6×
1E7×
>1.7E7×
Sequencing depth
Deletion
Combo mutation
c
Ranking within total AA (%)
1E4×
1E5×
1E6×
R78PSMB5
A79PSMB5
T80PSMB5
V90PSMB5
M104PSMB5
A108PSMB5
b
Sequencing depth (average base depth)
Deletion (fold change)
Combo mutation (fold change)
Substitution frequency
Amino Acid
18,000,000 ×
10,000 ×
100,000 ×
1,000,000 ×
Replicate 1
Replicate 2
Replicate 3

Figure S16 Correlation of NGS depth and data quality for PASTMUS of PSMB5. **a** Sequencing depth determination of PSMB5 library before screening. **b** a.a. deletion, combo mutation fold changes and a.a. substitution frequency corresponding to each a.a. at sequencing depths ranging from 1E4× to 1E6× of library after screening and 1E7× of library before screening. **c** Ranking of validated critical sites at corresponding sequencing depths.
