# Supplementary material for: PASTMUS: mapping functional elements at single amino acid resolution in human cells
Source: Genome Biol. 2019 Dec 16;20:279. doi: 10.1186/s13059-019-1897-7 (PMC6913009; doi:10.1186/s13059-019-1897-7)
Supplement: Supplementary file 5 — Additional file 5: Table S4. Primers used for sgRNA oligos amplification. Table S5. Primers used for cDNA amplification. Table S7. Primers used to generate different deletion mutants for HBEGF. Table S8. sgRNAs and ssODNs used for PSMB5 and PLK1 mutants validation. Table S9. Primers used for PSMB5 genome amplification. Table S10. Primers used to generate PSMB5 M104 and V90 mutants. Table S11. Summary of candidate validations. [file 13059_2019_1897_MOESM5_ESM.pdf]

Supplementary Tables

Supplementary Table 1 | Information of CRISPR mediated single site mutagenesis of *HBEGF* and *CSPG4*.

Supplementary Table 2 | Information of six genes used in PASTMUS.

Supplementary Table 3 | sgRNA sequences used for PASTMUS.

Supplementary Table 4 | Primers used for sgRNA oligos amplification.

Supplementary Table 5 | Primers used for cDNA amplification.

Supplementary Table 6 | Essential scores of amino acids from six proteins through PASTMUS.

Supplementary Table 7 | Primers used to generate different deletion mutants for *HBEGF*.

Supplementary Table 8 | sgRNAs and ssODNs used for *PSMB5* and *PLK1* mutants validation.

Supplementary Table 9 | Primers used for *PSMB5* geneome amplification.

Supplementary Table 10 | Primers used to generate PSMB5 M104 and V90 mutants.

Supplementary Table 11 | Summary of candidate validations.

Supplementary Table 4 | Primers used for sgRNA oligos amplification.

Primer	Sequence
F^{toxin}	5’-TCTTCATATCGTATCGTGCG-3’
R^{toxin}	5’-TAGTCGCTAGGCTATAACGT-3’
F^{drug}	5’-TTGTGGAAAGGACGAAACCG-3’
R^{drug}	5’-TGCTGTCTCTAGCTCTACGT-3’

Supplementary Table 5 | Primers used for cDNA amplification.

Gene	Primer	Sequence
ANTXR1 (Transcript 1)	F1^{ANTXR1}	5’-AACAGCATCGGAGCGGAAA-3’
	R1^{ANTXR1}	5’-TGGGCTTTATCACCACTCCTC-3’
ANTXR1 (Transcript 3)	F2^{ANTXR1}	5’-AATAAAGGACCCGCGAGGAAG-3’
	R2^{ANTXR1}	5’-TTTTCAGGAGTGTGCTGTCCG-3’
CSPG4	F1^{CSPG4}	5’-TCCCAGCTCCCAGGACTC-3’
	R1^{CSPG4}	5’-GGGTGTTCTGAGTGTGCAGT-3’
	F2^{CSPG4}	5’-AGAGAGCCACTGTGTGGATGC-3’
	R2^{CSPG4}	5’-GGAAGTGTGCTCGCCGTCAG-3’
	F3^{CSPG4}	5’-GGGCTCGTGCTGTTCTCAC-3’
	R3^{CSPG4}	5’-GCACCAGGCATGGAAGCAAT-3’
HBEGF	F1^{HBEGF}	5’-CGAAAGTGACTGGTGCCTCG-3’
	R1^{HBEGF}	5’-GGTCCCAATGGCAGATCCCT-3’
HPRT1	F1^{HPRT1}	5’-AGGCGAACCTCTCGGCTTT-3’
	R1^{HPRT1}	5’-CAATCCGCCCAAAGGGAAC-3’
PLK1	F1^{PLK1}	5’-CTCTGCTCGGATCGAGGTCT-3’
	R1^{PLK1}	5’-GATGCAGGTGGGAGTGAGG-3’
PSMB5 (Transcript 1 and 3)	F1^{PSMB5}	5’-TTCCCCGACCCCCTTCAGTG-3’
	R1^{PSMB5}	5’-AGGATGGGTCACTGTGTCCGT-3’
PSMB5 (Transcript 2)	F2^{PSMB5}	5’-TGGCCGACCTCACTTCC-3’
	R2^{PSMB5}	5’-AAGTAAAACAAATAGTCACCTCTGC-3’

Supplementary Table 7 | Primers used to generate different deletion mutants for HBEGF.

Name of Primers	Sequence
29Δ-F^{HBEGF}	5’-GACCGGAAAGTCCGTTTGCAAGAGGCAG-3’
29Δ-R^{HBEGF}	5’-CTAGCCCTCTCCGCCGCTCCAGGCTC-3’
63Δ-F^{HBEGF}	5’-GACCGGAAAGTCCGTTTGCAAGAGGCAG-3’
63Δ-R^{HBEGF}	5’-CTGCCTCTTGCAAACGGACTTTCCGGTC-3’
70Δ-F^{HBEGF}	5’-GCAAGAGGCAGATCTGCTTTTGAGAGTC-3’
70Δ-R^{HBEGF}	5’-GACTCTCAAAAGCAGATCTGCCTCTTGC-3’
115Δ-F^{HBEGF}	5’-CGGAAATACAAGGACTGCATCCATGGAG -3’
115Δ-R^{HBEGF}	5’-CTCCATGGATGCAGTCCTTGTATTTCCG -3’
119Δ-F^{HBEGF}	5’-GGACTTCTGCATCCATGAATGCAAATATGTG-3’
119Δ-R^{HBEGF}	5’-CACATATTTGCATTCATGGATGCAGAAGTCC -3’
125Δ-F^{HBEGF}	5’-GAATGCAAATATGTGGAGCTCCGGGCTCC-3’
125Δ-R^{HBEGF}	5’-GGAGCCCGGAGCTCCACATATTTGCATTC-3’
127Δ-F^{HBEGF}	5’-ATGTGAAGGAGCGGGCTCCCTCCTGC -3’
127Δ-R^{HBEGF}	5’-GCAGGAGGGAGCCCGCTCCTTCACAT-3’
133Δ-F^{HBEGF}	5’-GCTCCCTCCTGCTGCCACCCGGGTTAC -3’
133Δ-R^{HBEGF}	5’-GTAACCCGGGTGGCAGCAGGAGGGAGC -3’
134Δ-F^{HBEGF}	5’-CCCTCCTGCATCCACCCGGGTTACC -3’
134Δ-R^{HBEGF}	5’-GGTAACCCGGGTGGATGCAGGAGGG -3’
138Δ-F^{HBEGF}	5’-CTGCCACCCGGGTCATGGAGAGAGGTGTC-3’
138Δ-R^{HBEGF}	5’-GACACCTCTCTCCATGACCCGGGTGGCAG-3’
141Δ-F^{HBEGF}	5’-CCGGGTTACCATGGAAGGTGTCATGGGC-3’
141Δ-R^{HBEGF}	5’-GCCCATGACACCTTCCATGGTAACCCGG-3’
152Δ-F^{HBEGF}	5’-GCCTCCCAGTGGAACGCTTATATACCTATG-3’
152Δ-R^{HBEGF}	5’-CATAGGTATATAAGCGTTCCACTGGGAGGC-3’
153Δ-F^{HBEGF}	5’-CCTCCCAGTGGAAAATTTATATACCTATGACC-3’
153Δ-R^{HBEGF}	5’-GGTCATAGGTATATAAATTTTCCACTGGGAGG-3’

Supplementary Table 8 | sgRNAs and ssODNs used for PSMB5 and PLK1 mutants validation.

Gene	Amino acid	sgRNA	ssODN
PSMB5	R78	5'-GTAAGCACCCGCTGTAGCCC-3'	5'-TTTTTGTGGTCTTATGTGGCCTGTTTTGTGTTTTCCTCTGATCTTAACAGTTCCGCCATGGAGTCATAGTTGCAGCTGACAGCAACGCTACAGCGGGTGCTTACATTGCCTCCCAGACG-3'
PSMB5	A79	5'-GTAAGCACCCGCTGTAGCCC-3'	5'-TTTTTGTGGTCTTATGTGGCCTGTTTTGTGTTTTCCTCTGATCTTAACAGTTCCGCCATGGAGTCATAGTTGCAGCTGACAGCAGGACCACAGCGGGTGCTTACATTGCCTCCCAGACG-3'
PSMB5	T80	5'-GTAAGCACCCGCTGTAGCCC-3'	5'-TTTTTGTGGTCTTATGTGGCCTGTTTTGTGTTTTCCTCTGATCTTAACAGTTCCGCCATGGAGTCATAGTTGCAGCTGACAGCAGGGCTGCCGCGGGTGCTTACATTGCCTCCCAGACG-3'
PSMB5	V90	5'-CTATCACCTTCTTCACCGTC-3'	5'-TTTCCTCTGATCTTAACAGTTCCGCCATGGAGTCATAGTTGCAGCTGACTCCAGGGCTACAGCGGGTGCTTACATTGCCTCACAGACGGCCAAGAAGGTGATAGAGATCAACCCATACC-3'
PSMB5	M104	5'-CCTGCTAGGCACCATGGCTG-3'	5'-AGATGCGTTCCTTATTTCGAAGCTCATAGATTCGACATTGCCGAGCCAACAGCCGTTCCCAGAAGCTGCAATCCGCTGCGCCGCCAGCGATGGTGCCTAGCAGGTATGGGTTGATCTCT-3'
PSMB5	A108	5'-AATCCGCTGCGCCCCCAGCCA-3'	5'-ACTCCAGGGCTACAGCGGGTGCTTACATTGCCTCCCAGACGGTGAAGAAGGTGATAGAGATCAACCCATACCTGCTAGGCACAATGGCTGGGGGCACCGCGGATTGCAGCTTCTGGGAA-3'
PSMB5	D110	5'-GCGCAGCGGATTGCAGCTTC-3'	5'-CAGTTTGGAGGCAGCTGCTACAGAGATGCGTTCCTTATTTCGAAGCTCATAGATTCGACATTGCCGAGCCAACAGCCGTTCCCAGAAGCTGCAGGCCGCTGCGCCCCCAGCCATGGTGC-3'
PSMB5	C111	5'- GCGCAGCGGATTGCAGCTTC -3'	5'-CAGTTTGGAGGCAGCTGCTACAGAGATGCGTTCCTTATTTCGAAGCTCATAGATTCGACATTGCCGAGCCAACAGCCGTTCCCAGAAGCTGGCATCCGCTGCGCCCCCAGCCATGGTGC-3'
PSMB5	Negative	5'-TCTTAGCTGACTACGCGTAA-3'	5'-CGCAGCCTCGCCCACCAGCACGTCGTAGGATTCCACGGCTTTTTCGAGGACAACGACTTCGTGTTCGTGGTGTTGGAGCTCTGTAGCAGGGTGAGTGTCGCTGCTGGGGAACTGGAACT-3'
PLK1	C67	5'-GTCCGAGATCTCGAAGCACT-3'	5'-AAGAGATCCCGGAGGTCCTAGTGGACCCACGCAGCCGGCGGCGCTATGTGCGGGGCCGCTTTTTGGGCAAGGGCGGCTTTGCAAAGGTGTTCGAGATCTCGGACGCGGACACCAAGGAG-3'
PLK1	R136	5'-CAGCGACACTCACCCTCCGG-3'	5'-CAGCCTCGCCCACCAGCACGTCGTAGGATTCCACGGCTTTTTCGAGGACAACGACTTCGTGTTCGTGGTGTTGGAGCTCTGTAGGCGGGGCGTGAGTGTCGCTGCTGGGGAACTGGAAC-3'
PLK1	F183	5'-CCTTTTCCTGAATGAAGATC-3'	5'-CTCCCAGCCTCCTCCAAATTCCAGCCTCTTGTAGTGATGTCAAGCACCCCTGCAGGCTCAGCAACTCACCTATTTCACCTCGAGATCTTCATTCAGCAGAAGGTTGCCCAGCTTGAGG-3'
PLK1	Negative	5'-TCTTAGCTGACTACGCGTAA-3'	5'-ACTCCAGGGCTACAGCGGGTGCTTACATTGCCTCCCAGACGGTGAAGAAGGTGATAGAGATCAACCCATACCTGCTAGGCACAATGGCTGGGGGCGCGGATTGCAGCTTCTGGGAACGG-3'

Supplementary Table 9 | Primers used for PSMB5 genome amplification.

Name of Primers	Sequence	Description
F1$^{PSMB5\text{-}genome}$	5'-GTGTTTTTGTGGTCTTATGTGGCC-3'	For PCR amplification of sgRNA targeted region of *PSMB5* gene locus for Sanger sequencing (R78, T80, M104, A108).
R1$^{PSMB5\text{-}genome}$	5'-CATGTGGTTGCAGCTTAACTCAC-3'	
F2$^{PSMB5\text{-}genome}$	5'-GATGTGAAGCTCGGGTGACATT-3'	
R2$^{PSMB5\text{-}genome}$	5'-TCAGCATTGACACCAAGCCCTTT-3'	

Supplementary Table 10 | Primers used to generate PSMB5 M104 and V90 mutants.

Primer	Sequence
M104G-F	5’-ATAGAGATCAACCCATACCTGCTAGGCACCGGCGCTGGGGGCGCAGCGGATTG-3’
M104G-R	5’-CCAGAAGCTGCAATCCGCTGCGCCCCCAGCGCCGGTGCCTAGCAGGTATGGGT-3’
M104E-F	5’-ATAGAGATCAACCCATACCTGCTAGGCACCGAGGCTGGGGGCGCAGCGGATTG-3’
M104E-R	5’-CCAGAAGCTGCAATCCGCTGCGCCCCCAGCCTCGGTGCCTAGCAGGTATGGGT-3’
M104D-F	5’-ATAGAGATCAACCCATACCTGCTAGGCACCGACGCTGGGGGCGCAGCGGATTG-3’
M104D-R	5’-CCAGAAGCTGCAATCCGCTGCGCCCCCAGCGTCGGTGCCTAGCAGGTATGGGT-3’
M104V-F	5’-ATAGAGATCAACCCATACCTGCTAGGCACCGTGGCTGGGGGCGCAGCGGATTG-3’
M104V-R	5’-CCAGAAGCTGCAATCCGCTGCGCCCCCAGCCACGGTGCCTAGCAGGTATGGGT-3’
M104A-F	5’-ATAGAGATCAACCCATACCTGCTAGGCACCGCCGCTGGGGGCGCAGCGGATTG-3’
M104A-R	5’-CCAGAAGCTGCAATCCGCTGCGCCCCCAGCGGCGGTGCCTAGCAGGTATGGGT-3’
M104R-F	5’-ATAGAGATCAACCCATACCTGCTAGGCACCAGAGCTGGGGGCGCAGCGGATTG-3’
M104R-R	5’-CCAGAAGCTGCAATCCGCTGCGCCCCCAGCTCTGGTGCCTAGCAGGTATGGGT-3’
M104S-F	5’-ATAGAGATCAACCCATACCTGCTAGGCACCAGCGCTGGGGGCGCAGCGGATTG-3’
M104S-R	5’-CCAGAAGCTGCAATCCGCTGCGCCCCCAGCGCTGGTGCCTAGCAGGTATGGGT-3’
M104K-F	5’-ATAGAGATCAACCCATACCTGCTAGGCACCAAGGCTGGGGGCGCAGCGGATTG-3’
M104K-R	5’-CCAGAAGCTGCAATCCGCTGCGCCCCCAGCCTTGGTGCCTAGCAGGTATGGGT-3’
M104N-F	5’-ATAGAGATCAACCCATACCTGCTAGGCACCAACGCTGGGGGCGCAGCGGATTG-3’
M104N-R	5’-CCAGAAGCTGCAATCCGCTGCGCCCCCAGCGTTGGTGCCTAGCAGGTATGGGT-3’
M104M-F	5’-ATAGAGATCAACCCATACCTGCTAGGCACCATGGCTGGGGGCGCAGCGGATTG-3’
M104M-R	5’-CCAGAAGCTGCAATCCGCTGCGCCCCCAGCCATGGTGCCTAGCAGGTATGGGT-3’
M104T-F	5’-ATAGAGATCAACCCATACCTGCTAGGCACCACCGCTGGGGGCGCAGCGGATTG-3’
M104T-R	5’-CCAGAAGCTGCAATCCGCTGCGCCCCCAGCGGTGGTGCCTAGCAGGTATGGGT-3’
M104W-F	5’-ATAGAGATCAACCCATACCTGCTAGGCACCTGGGCTGGGGGCGCAGCGGATTG-3’
M104W-R	5’-CCAGAAGCTGCAATCCGCTGCGCCCCCAGCCCAGGTGCCTAGCAGGTATGGGT-3’
M104C-F	5’-ATAGAGATCAACCCATACCTGCTAGGCACCTGCGCTGGGGGCGCAGCGGATTG-3’
M104C-R	5’-CCAGAAGCTGCAATCCGCTGCGCCCCCAGCGCAGGTGCCTAGCAGGTATGGGT-3’
M104Y-F	5’-ATAGAGATCAACCCATACCTGCTAGGCACCTACGCTGGGGGCGCAGCGGATTG-3’
M104Y-R	5’-CCAGAAGCTGCAATCCGCTGCGCCCCCAGCGTAGGTGCCTAGCAGGTATGGGT-3’
M104F-F	5’-ATAGAGATCAACCCATACCTGCTAGGCACCTTCGCTGGGGGCGCAGCGGATTG-3’
M104F-R	5’-CCAGAAGCTGCAATCCGCTGCGCCCCCAGCGAAGGTGCCTAGCAGGTATGGGT-3’
M104Q-F	5’-ATAGAGATCAACCCATACCTGCTAGGCACCCAGGCTGGGGGCGCAGCGGATTG-3’
M104Q-R	5’-CCAGAAGCTGCAATCCGCTGCGCCCCCAGCCTGGGTGCCTAGCAGGTATGGGT-3’
M104H-F	5’-ATAGAGATCAACCCATACCTGCTAGGCACCCACGCTGGGGGCGCAGCGGATTG-3’
M104H-R	5’-CCAGAAGCTGCAATCCGCTGCGCCCCCAGCGTGGGTGCCTAGCAGGTATGGGT-3’
M104L-F	5’-ATAGAGATCAACCCATACCTGCTAGGCACCCTGGCTGGGGGCGCAGCGGATTG-3’
M104L-R	5’-CCAGAAGCTGCAATCCGCTGCGCCCCCAGCCAGGGTGCCTAGCAGGTATGGGT-3’
M104P-F	5’-ATAGAGATCAACCCATACCTGCTAGGCACCCCCGCTGGGGGCGCAGCGGATTG-3’
M104P-R	5’-CCAGAAGCTGCAATCCGCTGCGCCCCCAGCGGGGGTGCCTAGCAGGTATGGGT-3’
V90E-F	5’-GGGTGCTTACATTGCCTCCCAGACGGAGAAGAAGGTGATAGAGATCAA-3’
V90E-R	5’-TTGATCTCTATCACCTTCTTCTCCGTCTGGGAGGCAATGTAAGCACCC-3’

Supplementary Table 11 | Summary of candidate validations.

Protein target	Validated amino acid	Ranking in PASTMUS screening	Percentage of rank within total amino acids	Drug/toxin resistant in validation
HBEGF	L29	191st	91.38%	No
	D63	37th	17.70%	No
	D70	65th	31.10%	No
	F115	35th	16.74%	Yes
	G119	5th	2.39%	Yes
	K125	29th	13.88%	Yes
	L127	7th	3.35%	Yes
	I133	33rd	15.79%	Yes
	C134	36th	17.22%	Yes
	Y138	2nd	0.96%	Yes
	E141	22nd	10.53%	Yes
	N152	107th	51.19%	No
	R153	75th	35.88%	No
PLK1	C67	39th	6.46%	Yes
	R136	16th	2.64%	Yes
	F183	1st	0.17%	Yes
PSMB5	R78	4th	1.52%	Yes
	A79	2nd	0.76%	Yes
	T80	1st	0.38%	Yes
	V90	9th	3.41%	Yes
	M104	3rd	1.14%	Yes
	A108	10th	3.79%	Yes
	D110	149th	56.43%	No
	C111	150th	56.81%	No
